# Supplementary material for: Site-selective superassembly of biomimetic nanorobots enabling deep penetration into tumor with stiff stroma
Source: Nat Commun. 2023 Aug 2;14:4628. doi: 10.1038/s41467-023-40300-2 (PMC10397308; doi:10.1038/s41467-023-40300-2)
Supplement: Supplementary file 1 — Supplementary Information File [file 41467_2023_40300_MOESM1_ESM.pdf]

Supplementary information

Site-Selective Superassembly of Biomimetic Nanorobots Enabling Deep Penetration into Tumor with Stiff Stroma

Miao Yan[1,11], Qing Chen[2,11], Tianyi Liu[1], Xiaofeng Li[3], Peng Pei[1], Lei Zhou[2], Shan Zhou[1], Runhao Zhang[1], Kang Liang[4], Jian Dong[2], Xunbin Wei[5], Jinqiang Wang[6], Osamu Terasaki[7], Pu Chen[8], Zhen Gu[6], Libo Jiang[2]*, and Biao Kong[1,9,10]*

[1]Department of Chemistry, Shanghai Key Lab of Molecular Catalysis and Innovative Materials, *i*ChEM, Fudan University, Shanghai 200438, P. R. China

[2]Department of Orthopaedic Surgery, Zhongshan Hospital, Fudan University, No. 180 Fenglin Road, Shanghai 200032, P. R. China

[3]Department of Chemistry, The University of Hong Kong, Hong Kong 999077, P. R. China

[4]School of Chemical Engineering and Graduate School of Biomedical Engineering, The University of New South Wales, NSW 2052, Australia

[5]Biomedical Engineering Department and Cancer Hospital and Institute, Key Laboratory of Carcinogenesis and Translational Research, Peking University, Beijing 100081, P. R. China

[6]Key Laboratory of Advanced Drug Delivery Systems of Zhejiang Province, College of Pharmaceutical Sciences, Zhejiang University, Hangzhou 310063, P. R. China

[7]School of Physical Science and Technology, ShanghaiTech University, Shanghai 201210, P. R. China

[8]Department of Chemical Engineering, University of Waterloo, Waterloo, Ontario N2L 3G1, Canada

[9]Yiwu Research Institute of Fudan University, Yiwu, Zhejiang 322000, P. R. China

[10]Shandong Research Institute, Fudan University, Jinan, Shandong 250103, P. R. China

[11]These authors contributed equally: Miao Yan and Qing Chen

e-mail: jiang.libo@zs-hospital.sh.cn; bkong@fudan.edu.cn

1. Instruments

Transmission electron microscopy (TEM) images were obtained with a JEOL 2011F microscope (Japan) operating at 200 kV. Scanning electron microscopy (SEM) images were recorded by a Hitachi S-4800 ultrahigh resolution cold FEG with an in-lens electron optic operating at 20 kV. UV-vis-NIR spectra were collected using a HITACHI U-4100 (Tokyo, Japan). The EDS elemental mapping was carried out by a Tecnai G2 F20 S-Twin STEM (FEI, USA). Laser irradiation experiments were performed by employing a diode laser with a wavelength of 980 nm. The photothermal effects were recorded using a T460SC infrared thermal imaging camera (FLIR, Sweden). Thermal gravimetric analysis (TGA) was carried out to characterize the urchin head/hollow tail nanostructures (UHHTNs) loaded with PCM at a rate of 10°C/min to 500°C in a nitrogen atmosphere. Differential scanning calorimetry (DSC) was conducted to test the thermal properties of PCM heated at a rate of 2°C per min under a nitrogen atmosphere. An Olympus FV3000 fluorescence microscope (Olympus, Japan) and a 980 nm fiber-coupled diode laser system (CNI Laser, MDL-III-980) were employed to record the motion of the nanorobots.

2. Supplementary experimental procedures

2.1. PCM, drug loading and in vitro drug release

In a typical process, 100 mg of lauric acid and 25 mg of stearic acid was dissolved in 0.5 mL of DMSO, followed by the addition of 50 mg of DOX under magnetic stirring for 30 min. Afterward, 20 mg of UHHTN precipitate (with an opening of 100 nm in

diameter) was redispersed in the above mixture under stirring overnight at 25°C. The obtained suspension was then kept in a vacuum oven for 10 min to remove the air trapped in the hollow interior of the UHHTNs. During this process, the mixture of PCM and DOX slowly entered the cavity of each UHHTN through the opening on the tail. Finally, the UHHTNs loaded with the PCM and DOX suspension were collected by by centrifugation at (3300 rmp, centrifugal force, 803 g) for 7 min, and the supernatant solution was removed. The excess fatty acids and DOX was removed after another two rounds of washing with DMSO. Afterward, 1mL of deionized water was added to the above precipitate to solidify the PCM and retain the DOX inside the cavity of the UHHTNs.

Note: To avoid forming DOX-trapped in lipid self-assembled nanoparticles, two rounds of washing with DMSO are required to remove PCM adsorbed on the outer surface of UHHTNs, unloaded fatty acids and free DOX in solution. Then water is added to solidify the DOX-trapped fatty acids inside the cavity. This process ensures that resulting final products are only DOX-trapped UHHTN nanostructures.

The DOX loading amount and its release profile were examined by UV-vis spectroscopy. To assess the drug loading performance of the UHHTNs, the amount of DOX loaded on the UHHTNs was quantified with the assistance of standard concentration curve of DOX at the absorption of 480 nm, which is generated by DOX solution with different concentrations.

The drug loading capacity of the UHHTNs can be calculated through the equation below:

$$\text{Loading capacity} = \frac{\text{Mass of drug loaded (mg)}}{\text{Mass of UHHTNs (mg)}}$$

"The NIR-triggered release profile of DOX from nanoparticles was performed in DMEM, pH 7.4. At defined time periods, release media was collected to detect the content of drugs. To testify that the laser irradiation can accelerate the drug release, the same amount of drug-loaded NPs was added in DMEM without NIR laser irradiation at the indicated time points. The release profiles of DOX were analyzed by UV-Vis spectrometer post purification at different intervals.

2.2. Synthesis of Cy5.5 labelled UHHTN/DOX and AuNS@SiO_2 nanoparticles

First, the Cy5.5 conjugation with APTES was performed. Cy5.5-NHS and 10 μL APTES was dispersed in 500 μL of DMSO. The mixture was then stirred for 24 h at room temperature in a dark environment. Then 50 μL of Cy5.5-APTES solution was added 2 mL of ethanol solution with UHHTN/DOX and AuNS@SiO_2, followed by stirring for 12 h under a dark environment. The Cy5.5 was successfully conjugated onto the nanoparticles.

2.3. Record and track the motion of nanorobots

To investigate UHHTN motion performance, visualization of nanorobots was performed using an optical microscope (63X, oil objective) mounted on an isolation

table and equipped with a NIR laser system to supply the 980 nm NIR light to trigger the motion and a digital camera to record the motion frames of the nanorobots.

First, 1 μL of nanoparticles was mixed into 200 μL of DMEM with 10% serum and then the samples were filled in a borosilicate capillary and sealed with capillary wax (Hampton Research) for minimizing the drifting effect. The capillary filled with sample was then attached to a glass slide (thickness = 1 mm) for observation under an inverted optical microscope with an oil immersion lens and high numerical aperture objectives. Finally, Optical videos were achieved by a CCD camera. A movie at a rate of 40 frames per second was recorded for each particle.

With a suitable video clip recorded, the first step is to extract the positional information on nanorobots with a tracking program. The resulting movie clips of the particles are analyzed using particle tracking method by Image J software. We used a Labview (National Instruments) script to obtain particle trajectory videos. In brief, a computer algorithm finds the position of nanorobots in movies clips being tracked. As nanorobots appear as dark circles in the movie clips, and the background as light gray, the script can detect them by applying a threshold to each frame of the movie. Then we use algorithms built-in to the LabView software to find circular objects in these binary images. Once these nanorobots are found, another built-in algorithm finds the centers of these circles and records their x,y-coordinates along with the time, the frame was captured (relative to the movie clip length). Finally, following particle tracking, the script outputs a spreadsheet with its x and y coordinates at each time step to a comma separated value file (.CSV) for data storage. These data are then used to calculate its

mean squared displacement. For each NIR laser power approximately 10 separate particles were tracked and analyzed.

Calculating the Mean Squared Displacement

A more appropriate way of quantifying the mobility of a nanorobot is to calculate its Mean Squared Displacement (MSD) ($\langle \Delta L^2 \rangle$). In brief, one takes the average value of the square of the displacement (ΔL^2) within a certain time interval Δt and plots the averages ($\langle \Delta L^2 \rangle$) over increasing Δt. ΔL represents the displacement of a nanorobots in a time interval Δt. The resulting video clip with coordinates of the nanorobots versus time can be used to calculate them. The general principle of calculating MSD has been amply explained in the literature[1].

Analyzing the Mean Squared Displacement

With the generation of an MSD plot, we then analyze and extract the mobility of the nanorobots. A diffusive system would show a linear component according to equation 1 from which diffusion coefficient D can be extracted. For nanorobots with 0-1.5 W·cm^{-2} NIR lase irradiation, the diffusion coefficient is obtained by the linear fitting of MSD versus time interval (Δt) by applying equation 1.

$$\mathrm{MSD}(\Delta t) = 2d \cdot D_t \cdot \Delta t \qquad (1)$$

When MSD curves show a parabolic fit according to the equation 3 from which we can extract the diffusivity D of the particles. This extraction principle has been amply explained in the literature[2].

The diffusion coefficient of the nanorobots with 2.0 W·cm^{-2} NIR lase irradiation was produced according to equation 2.

$$\mathrm{MSD}(\Delta t) = 2d \cdot D_t \cdot \Delta t + (V \cdot \Delta t)^2 \qquad (2)$$

(where d = dimensionality (2 in our case for the NTA measurements));

3. Supplementary Figures

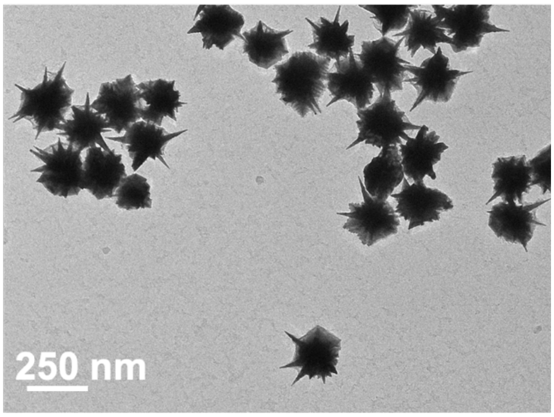

Supplementary Fig. 1. Morphological characterization. TEM image of nanostructures trapped at reaction times of 0.5 h. Experiment was repeated three times independently with similar results.

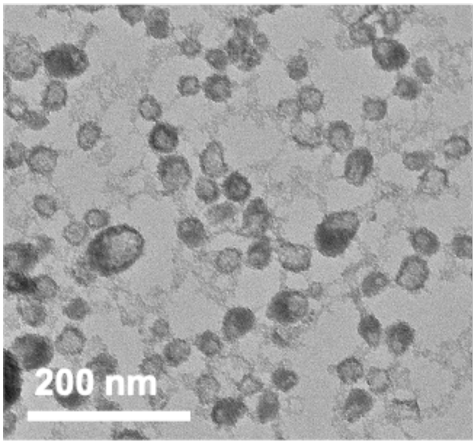

Supplementary Fig. 2. Morphological characterization. TEM image of self-nucleated hollow silica nanoparticles formed in solution and obtained through centrifugation at a higher rate. Experiment was repeated three times independently with similar results.

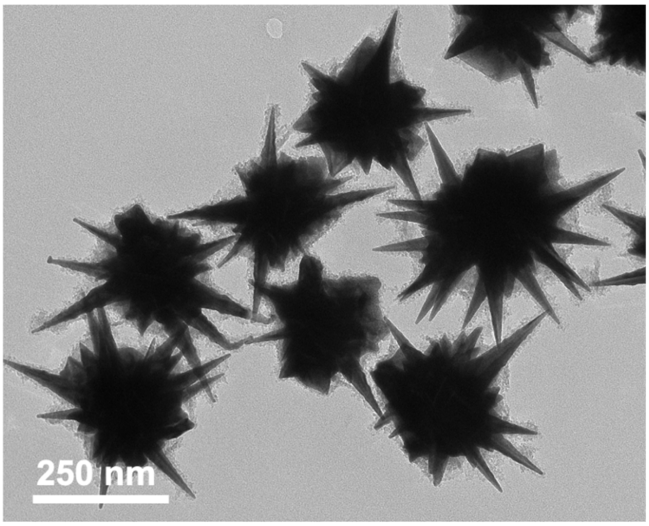

Supplementary Fig. 3. Morphological characterization. TEM image of nanostructures obtained at reaction times of 0.5 h. Experiment was repeated three times independently with similar results.

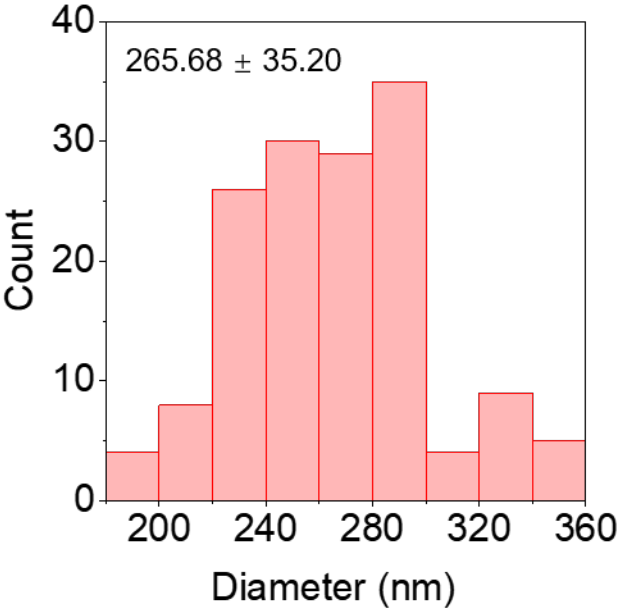

Supplementary Fig. 4. Diameter distribution of AuNSs determined by TEM (150 AuNSs analyzed).

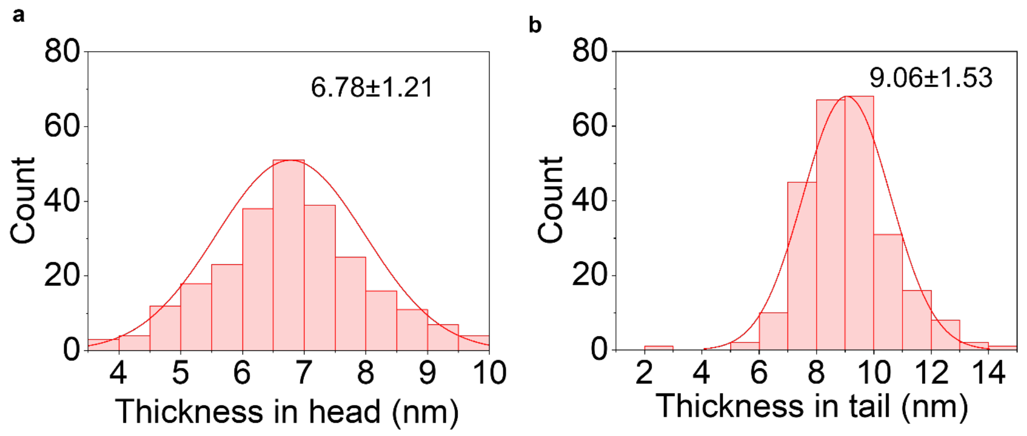

Supplementary Fig. 5. The statistic analysis of the thickness in head and tail of UHHTNs. Histogram showing the distribution of the silica shell thickness in head (a) and tail region (b) of resulting UHHTNs.

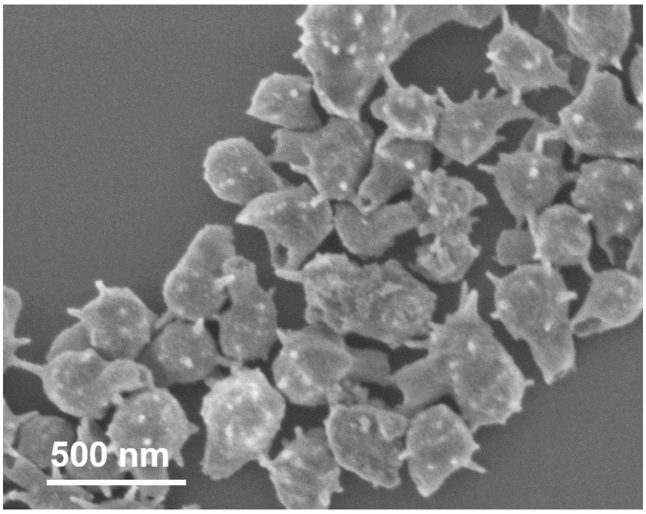

Supplementary Fig. 6. SEM image of the UHHTNs obtained after dispersing the nanoparticles in ethanol and sonicating for 2 h. The morphologies of the nanocomposites showed intact structure, indicating the excellent stability. Experiment was repeated three times independently with similar results.

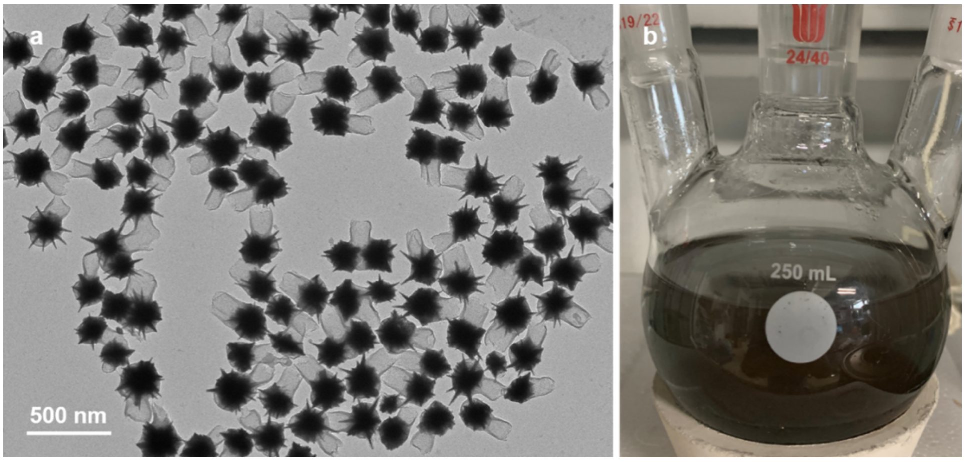

Supplementary Fig. 7. Morphological characterization of UHHTNs in a large-scale synthesis process. (a) TEM image of UHHTNs and (b) a digital photo of the 250 mL reaction vessel showing the product of a scaled-up synthesis (~182 mL). For the scaled-up synthesis of UHHTNs, all the amounts of the corresponding chemicals and AuNSs used were multiplied by 50 and the reaction was carried out in a 250 mL round-bottomed flask. The whole process remained the same as in the small amount synthesis. Experiment was repeated three times independently with similar results.

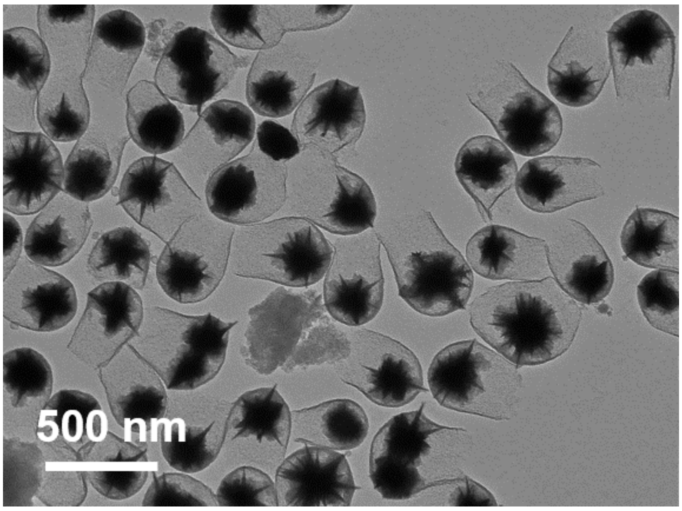

Supplementary Fig. 8. Morphological characterization. TEM image of nanostructures prepared at 5 mM 4-MPAA concentration. Experiment was repeated three times independently with similar results.

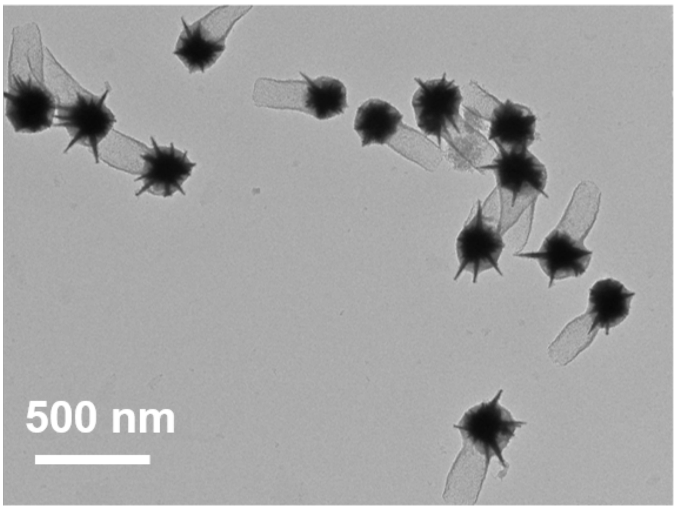

Supplementary Fig. 9. Morphological characterization. TEM image of nanostructures prepared at 15 mM 4-MPAA concentration. Experiment was repeated three times independently with similar results.

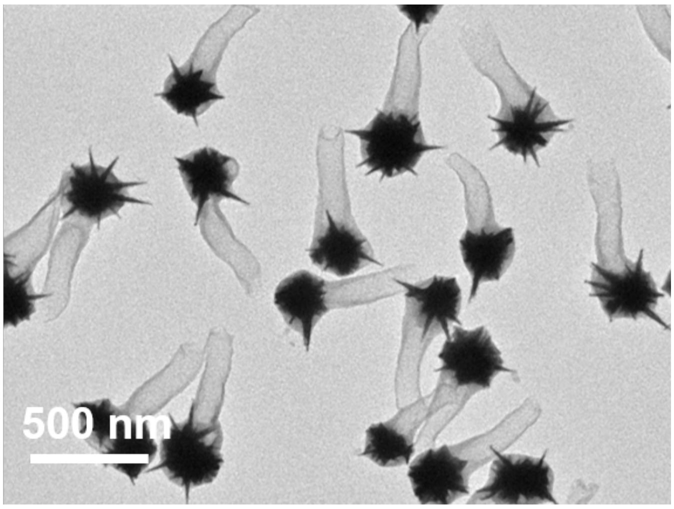

Supplementary Fig. 10. Morphological characterization. TEM image of nanostructures prepared at 25 mM 4-MPAA concentration. Experiment was repeated three times independently with similar results.

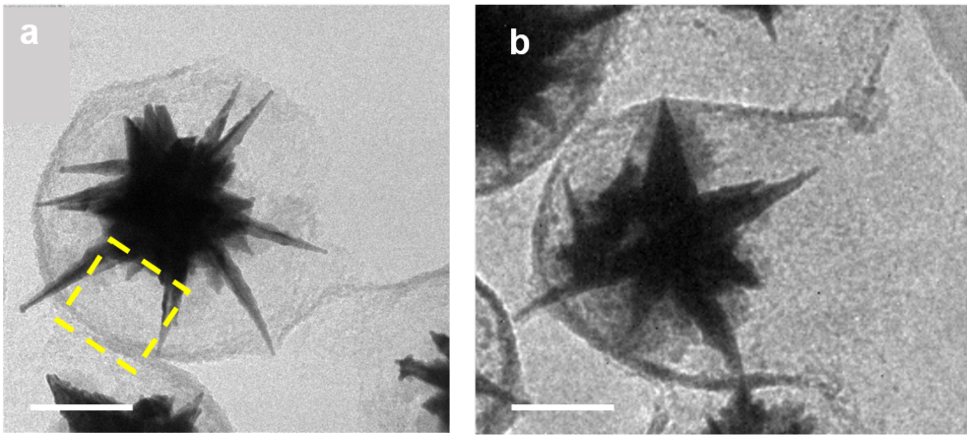

Supplementary Fig. 11. Growth process of the UHHTNs at a low 4-MPAA concentration. TEM images of the growth intermediates of UHHTNs trapped at different reaction times of (a) 0.5 h and (b) 4 h. Scale bars represent 100 nm. When t = 0.5 h, a thin silica layer formed along the tip end of the 4-MPAA-coated AuNS surface. A large hollow cavity length in the head can be observed (indicated by the yellow dotted box). Prolonging the growth time allowed silica to further deposit forward along the outermost layer of the PAA, giving short tube-like protrusions. Experiment was repeated three times independently with similar results.

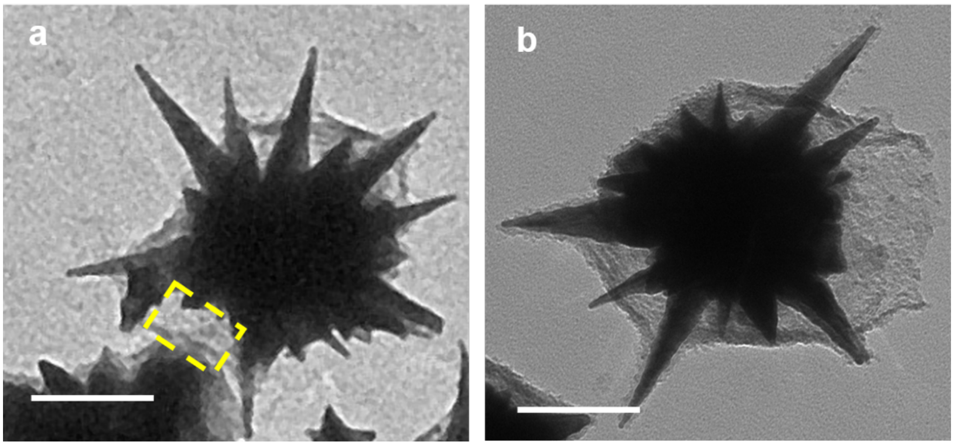

Supplementary Fig. 12. Growth process of the UHHTNs at a moderate 4-MPAA concentration. TEM images of the growth intermediates of UHHTNs trapped at different reaction times of (a) 0.5 h and (b) 2 h. Scale bars represent 100 nm. When t = 0.5 h, a coarse thin silica layer formed along the middle of the tip and groove in the 4-MPAA-coated AuNS surface, giving a smaller cavity length in thehead (indicated by the yellow dotted box). Prolonging the growth time allowed the silica to further deposit forward along the outermost layer of the PAA, giving short tube-like protrusions. Experiment was repeated three times independently with similar results.

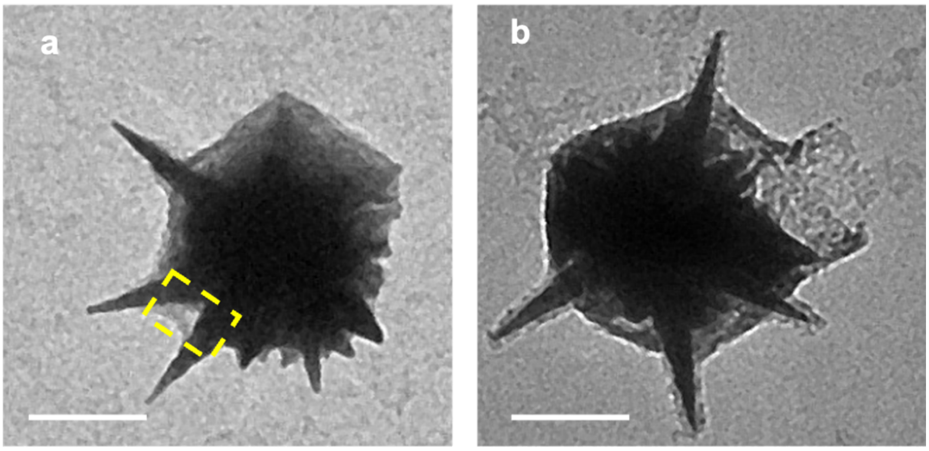

Supplementary Fig. 13. Growth process of the UHHTNs at high 4-MPAA concentration. TEM images of the growth intermediates of UHHTNs trapped at different reaction times of (a) 0.5 h and (b) 2 h. Scale bars represent 100 nm. When t = 0.5 h, a coarse thin silica layer formed along the spiky topology of the 4-MPAA-coated AuNS surface. The cavity in the head (indicated yellow dotted box) in the head almost disappeared. Prolonging the growth time allowed the silica to further deposit forward along the outermost layer of the PAA, giving short tube-like protrusions. Experiment was repeated three times independently with similar results.

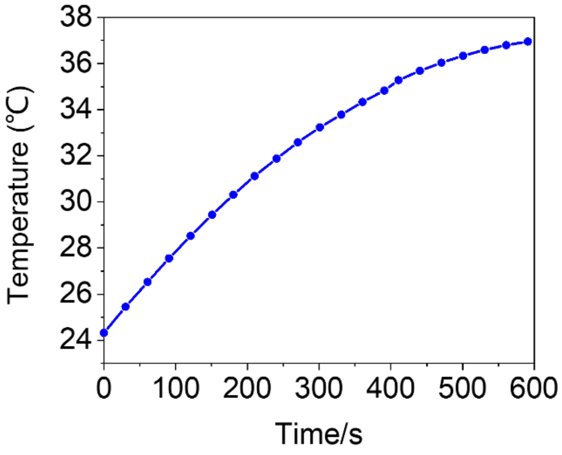

Supplementary Fig. 14. Photothermal curve of the pure water solution. Pure water solution was irradiated with 980 nm laser irradiation at 2 W·cm^{-2} for 10 min, and the temperature of the pure water solution increased to 37°C.

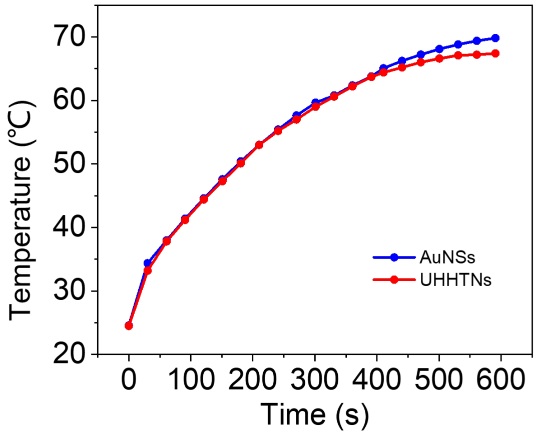

Supplementary Fig. 15. Photothermal curves of bare AuNS aqueous solutions and UHHTN aqueous solution under 980 nm irradiation at 2 W·cm^{-2} for 10 min show that the photothermal effects of the two kinds of nanoparticles are almost the same, indicating that the silica shell does not affect their photothermal conversion efficiency.

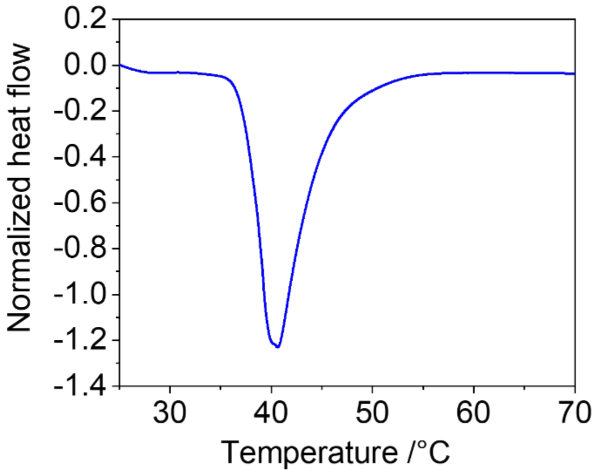

Supplementary Fig. 16. DSC curve of UHHTNs loaded with pure PCM.

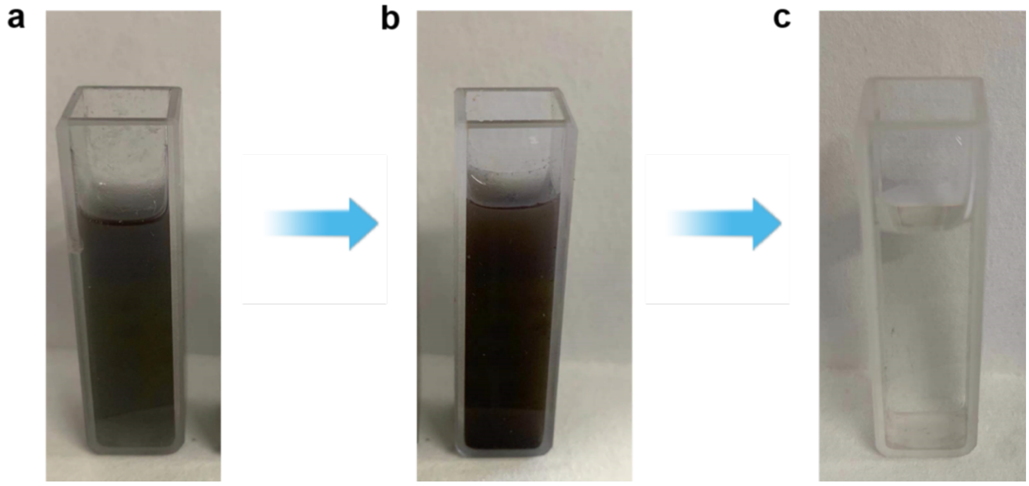

Supplementary Fig. 17. Visual color change before and after DOX loading. Photographs of (a) UHHTN solution before loading, (b) PCM/DOX-loaded UHHTN solution, and (c) supernatant after centrifugation of PCM/DOX-loaded UHHTNs solution. It was found that there is obvious color difference between a and b, and the DOX-loaded UHHTNs solution showed little red color. To further confirm that the red color was attributable to the DOX inside the cavity rather than to free DOX in solution, solution b was then centrifuged, and the resulting supernatant was nearly colorless, indicating the successful encapsulation of DOX.

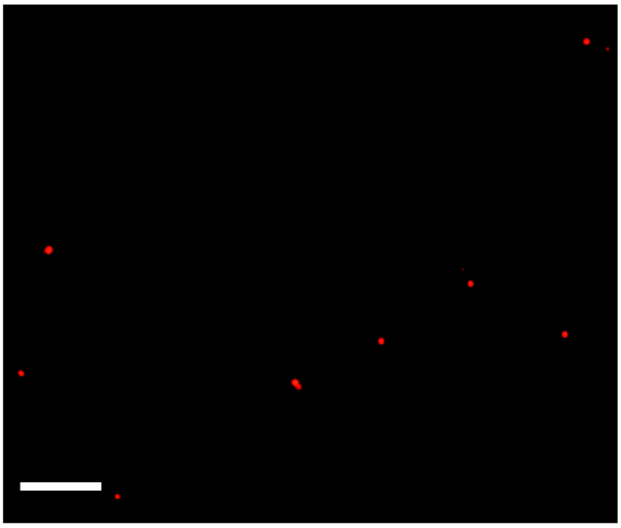

Supplementary Fig. 18. The fluorescence microscopy images of UHHTNs loaded with PCM/DOX. Scale bar, 5 μm. Experiment was repeated three times independently with similar results.

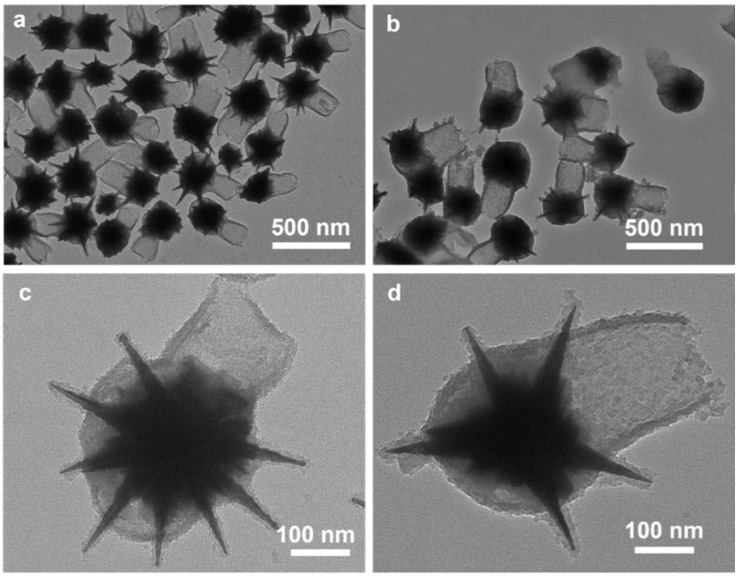

Supplementary Fig. 19. TEM images of the UHHTNs before (a and c) and after loading PCM/DOX (b and d). Compared with the UHHTNs without loading PCM/DOX, the tail interior of PCM/DOX-loaded UHHTNs showed a noticeable increase in contrast when observed under TEM, confirming that the PCM-DOX were loaded into the tails. Experiment was repeated three times independently with similar results.

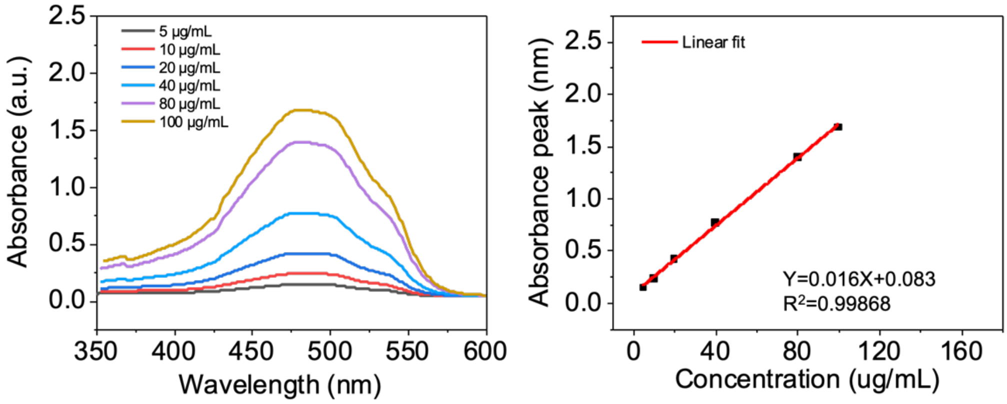

Supplementary Fig. 20. Absorption spectra of DOX in DMEM with 10% serum medium at various concentrations (left). Fitting curve of the absorption values at 480 nm of DOX solution at different concentrations (right).

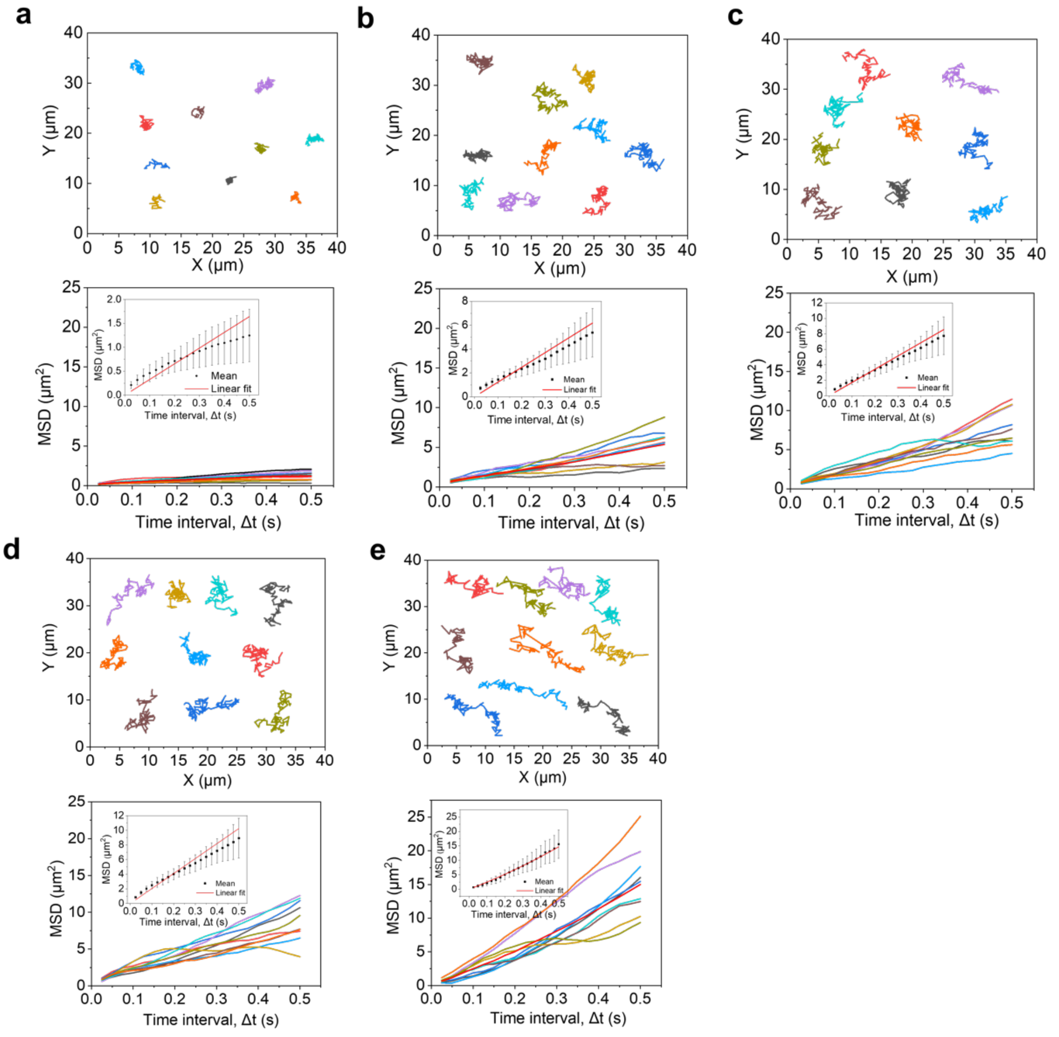

Supplementary Fig. 21. Movement performance. Tracking trajectories (top) and their corresponding calculated MSD functions (colors match) (bottom) of randomly selected UHHTNs upon NIR irradiation with different power densities: (a) 0 W cm^{-2}, (b) 0.5 W cm^{-2}, (c) 1.0 W cm^{-2}, (d) 1.5 W cm^{-2} and (e) 2.0 W cm^{-2}. Inset shows average MSD. Experimental data are mean ± s.d. (n = 10 independent samples). Source data are provided as a Source Data file.

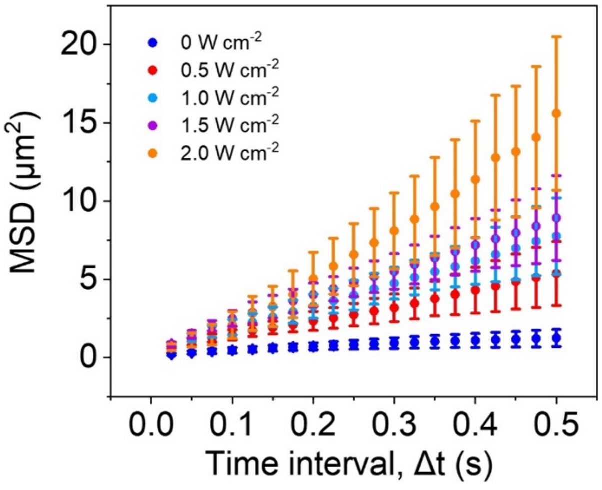

Supplementary Fig. 22. The average MSD of 10 randomly selected UHHTNs upon NIR irradiation with different power densities. Experimental data are mean ± s.d. of samples in a representative experiment (n = 10). Source data are provided as a Source Data file.

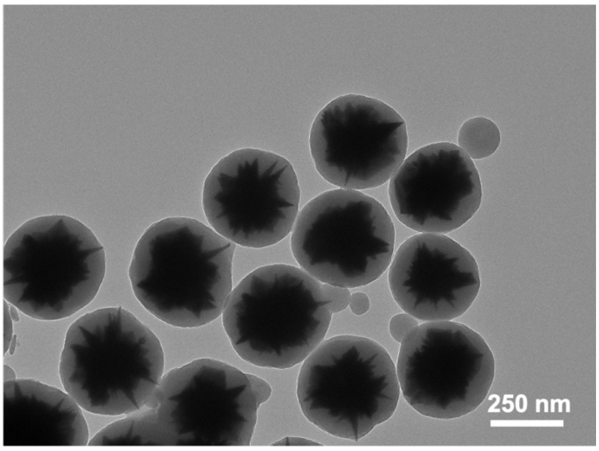

Supplementary Fig. 23. TEM image of AuNS@SiO_2 core-shell structured nanoparticle with an average size of ~350 nm. Experiment was repeated three times independently with similar results.

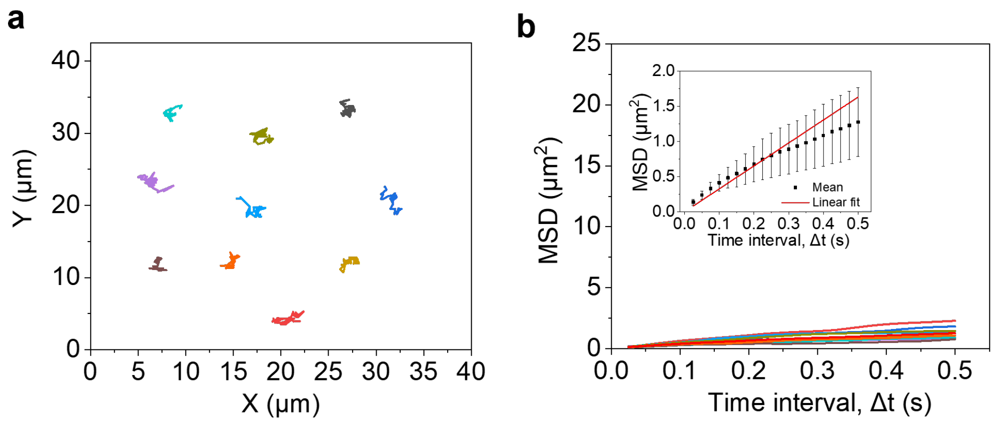

Supplementary Fig. 24. Movement performance of AuNS@SiO_2 core-shell structured nanoparticle. Tracking trajectories (a) and their corresponding calculated MSD functions (b) (colors match) (bottom) of randomly selected UHHTNs upon NIR irradiation at 0 W cm^{-2} power densities. Experimental data are mean ± s.d. of samples in a representative experiment (n = 10). Source data are provided as a Source Data file.

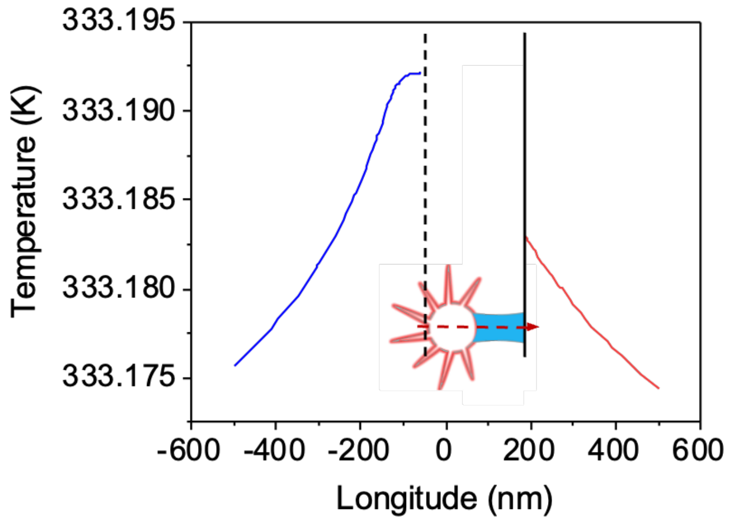

Supplementary Fig. 25. The computed temperature distribution of a PCM/nanorobot.

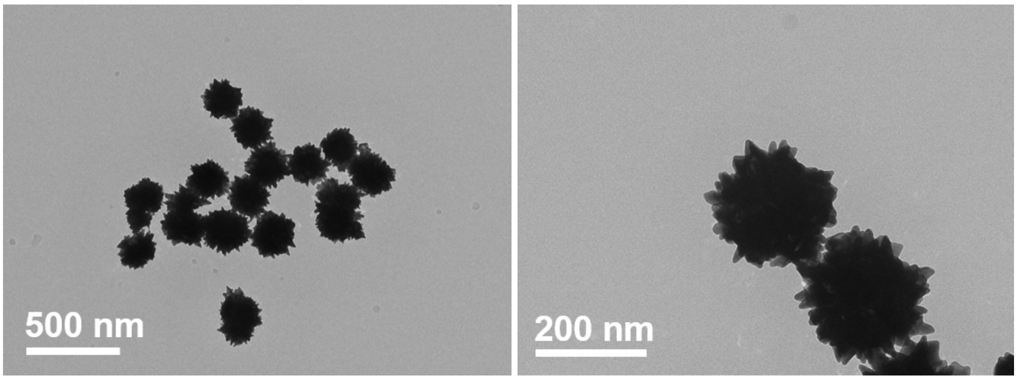

Supplementary Fig. 26. Morphological characterization of Au nanoparticles with short protrusions. The TEM images of Au nanoparticles with short protrusions as initial seeds. Experiment was repeated three times independently with similar results.

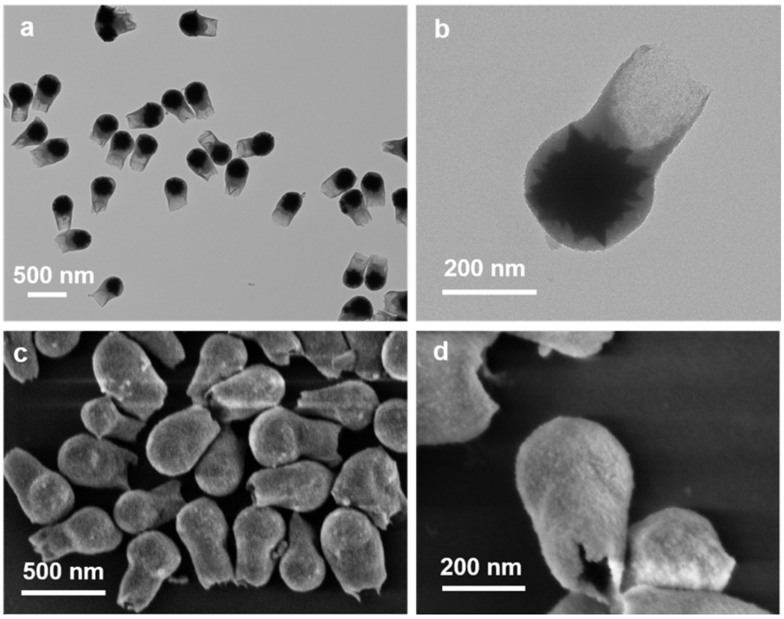

Supplementary Fig. 27. Morphological characterization of nanostructures with smooth surfaces. (a, b) The TEM images and (c, d) SEM images of nanostructures with smooth surfaces. Experiment was repeated three times independently with similar results.

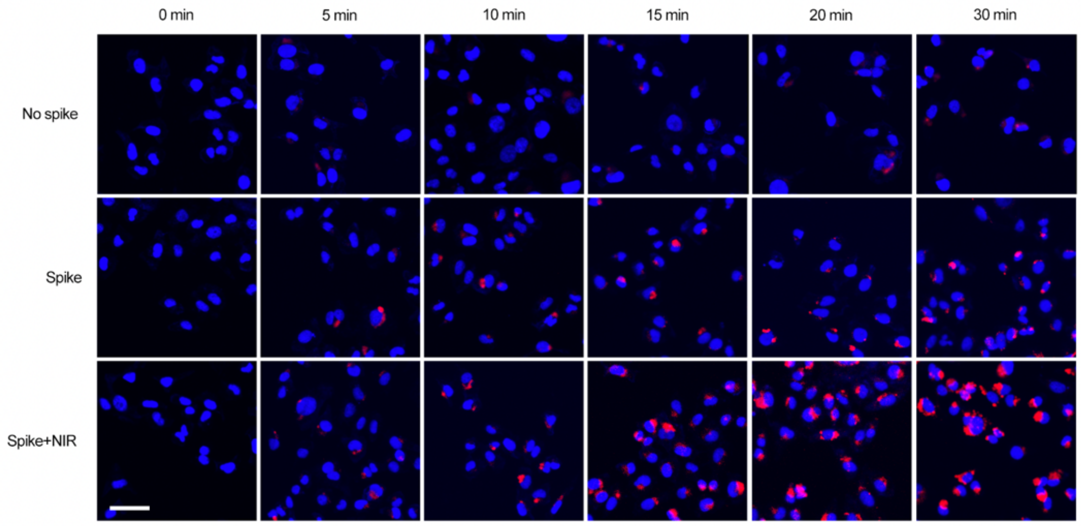

Supplementary Fig. 28. Fluorescence image of nanoparticles internalization in MDA-MB-231 tumor cells. Internalization of nanoparticles without nanospikes, spiky nanoparticles and spiky nanorobots in MDA-MB-231 tumor cells after 0, 5, 10, 15, 20 and 30 min of intervention. Scale bar, 25 μm.

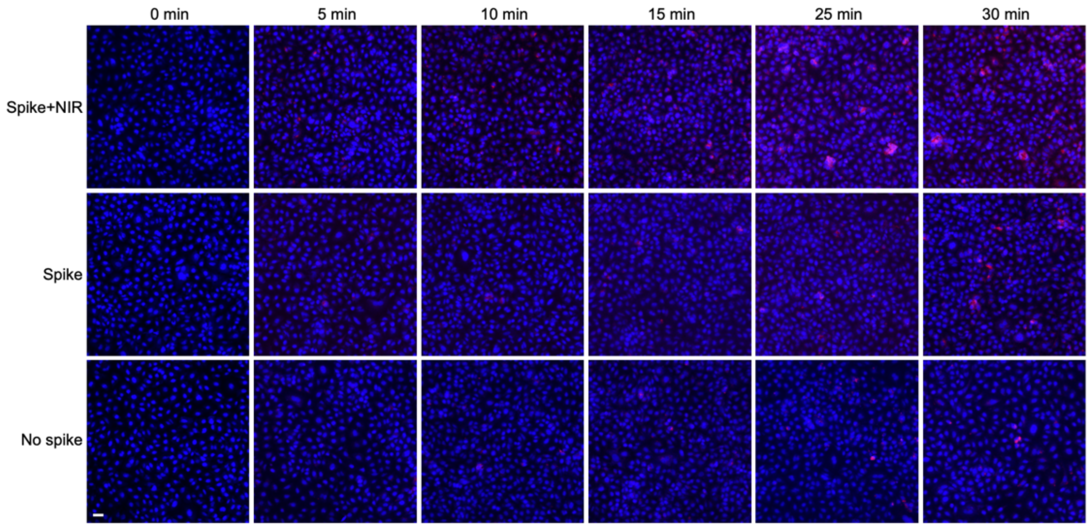

Supplementary Fig. 29. Fluorescence image of nanoparticles internalization in A549 tumor cells. Internalization of nanoparticles without nanospikes, spiky nanoparticles and spiky nanorobots in A549 tumor cells at different time points. Scale bar, 25 μm.

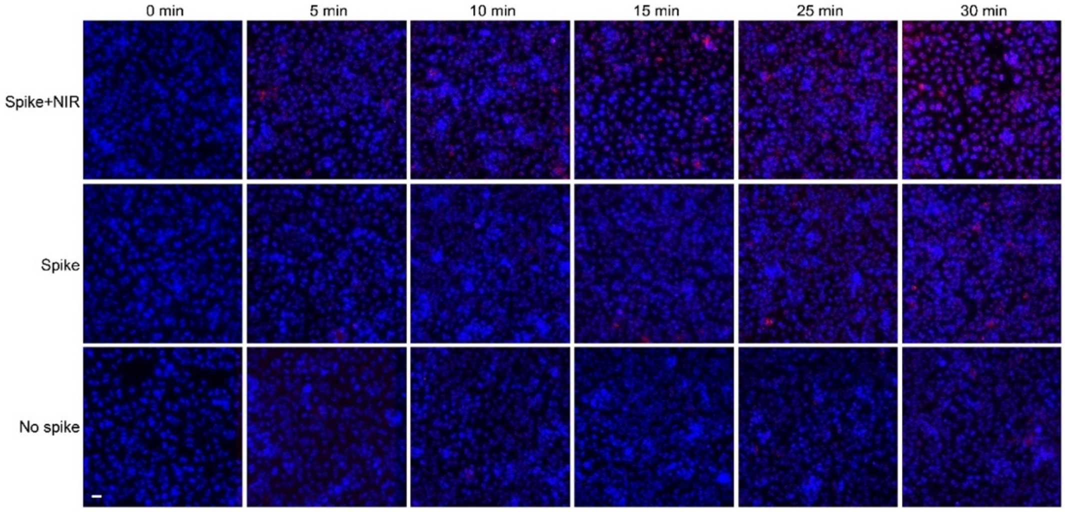

Supplementary Fig. 30. Fluorescence image of nanoparticles internalization in PANC-1 tumor cells. Internalization of nanoparticles without nanospikes, spiky nanoparticles and spiky nanorobots in PANC-1 tumor cells at different time points. Scale bar, 25 μm.

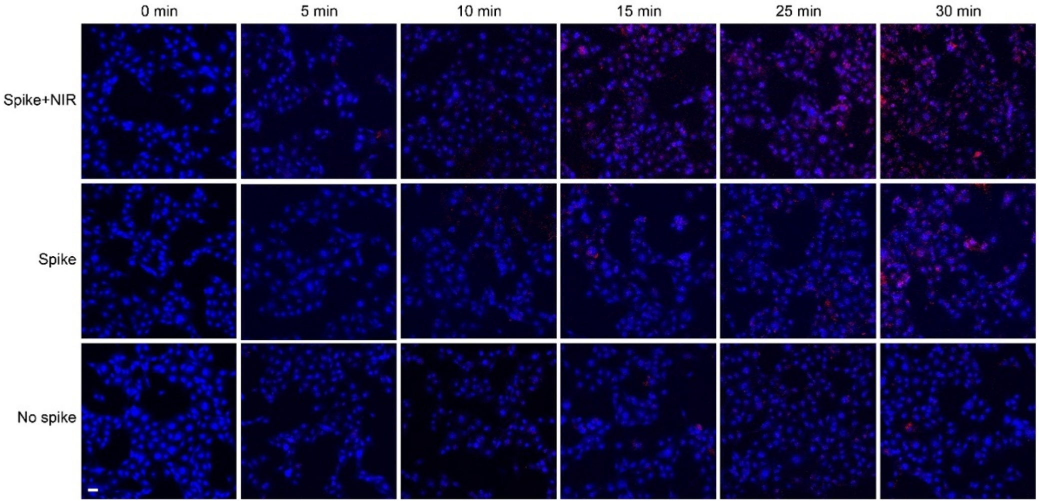

Supplementary Fig. 31. Fluorescence image of nanoparticles internalization in B16F10 tumor cells. Internalization of nanoparticles without nanospikes, spiky nanoparticles and spiky nanorobots in B16F10 tumor cells at different time points. Scale bar, 25 μm.

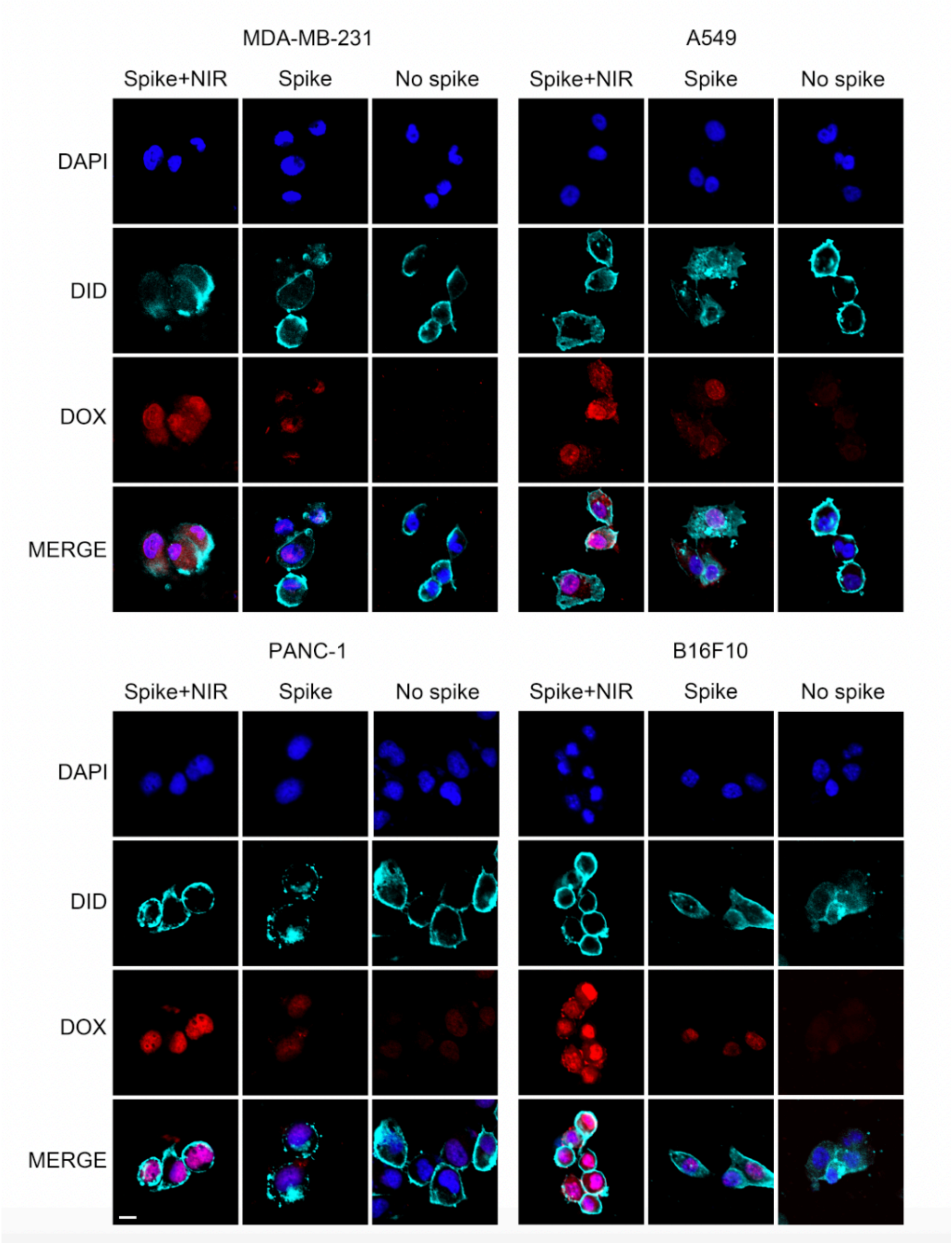

Supplementary Fig. 32. Fluorescence image of nanoparticles internalization in different tumor cells. Internalization of nanoparticles without nanospikes, spiky nanoparticles and spiky nanorobots in four tumor cells (MDA-MB-231, A549, PANC-1 and B16F10) at 30 min. Nuclei were stained with DAPI (blue). Cell membranes were stained with DID (sky blue). Fluorescent signal of DOX is shown in red. Scale bar, 10 µm. 1,1'-Dioctadecyl-3,3,3',3'-Tetramethylindodicarbocyanine, DID 4-Chlorobenzenesulfonate Salt; 4,6-diamidino-2-phenylindole, DAPI.

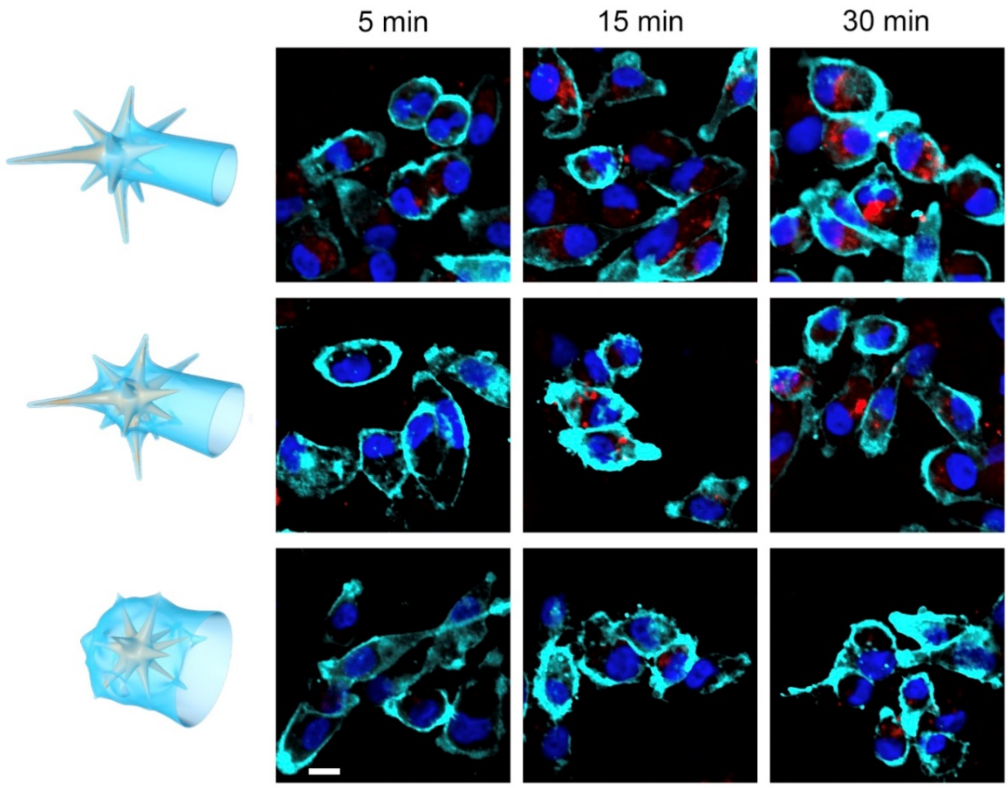

Supplementary Fig. 33. Fluorescence graphs of different nanoparticles internalization. Confocal fluorescence map of MDA-MB-231 human breast cancer cells with three spike lengths of UHHTNs over 30 min. Nuclei were stained with DAPI (blue). Cell membranes were stained with DID (sky blue), and fluorescent signal of DOX is shown in red. Scale bar, 10 μm.

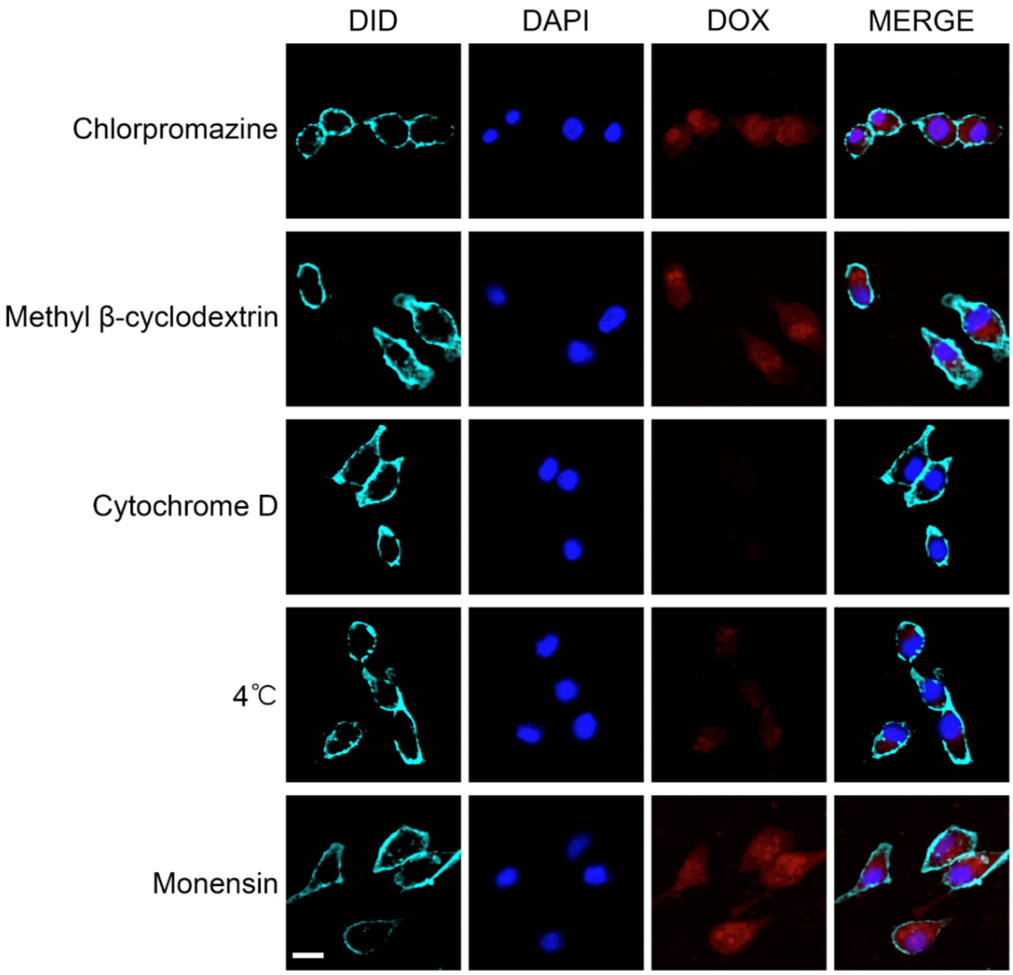

Supplementary Fig. 34. Fluorescence graph of nanorobots internalization pathway. CLSM images of UHHTN nanorobots irradiated in NIR in the presence of different inhibitors. Nuclei were stained with DAPI (blue). Cell membranes were stained with DID (sky blue), and fluorescent signal of DOX is shown in red. Scale bar, 15 μm.

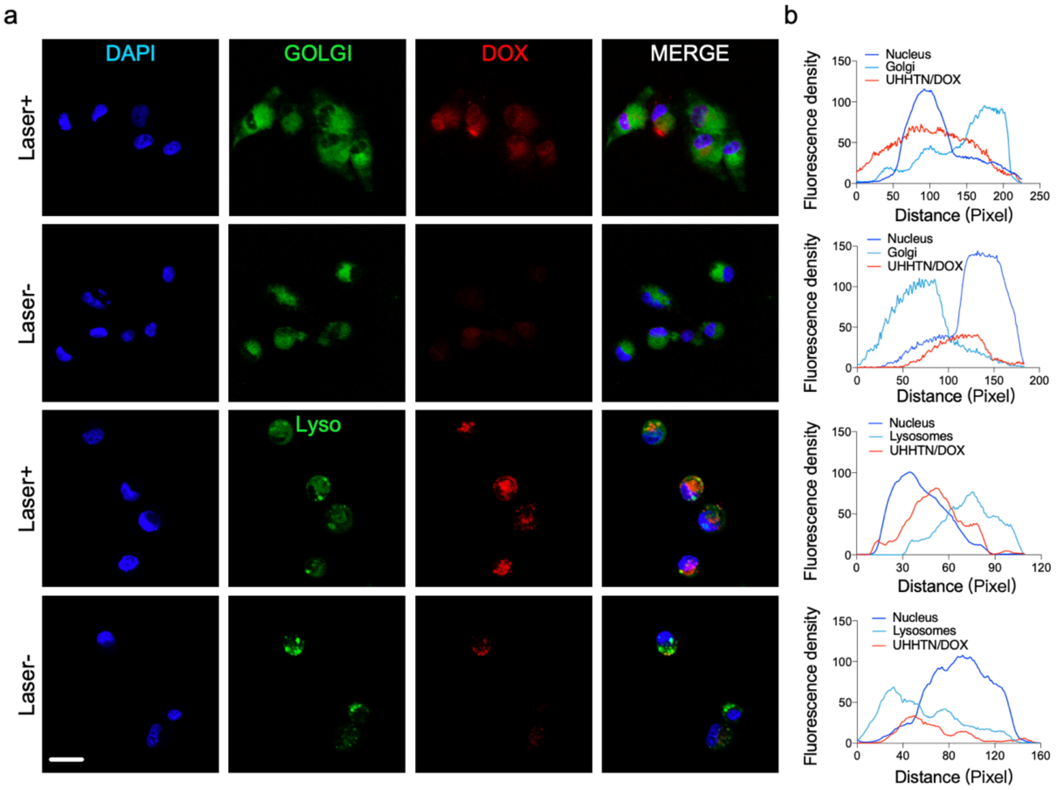

Supplementary Fig. 35. Fluorescence graph of nanoparticles internalization localization. (a) Co-localization of nanorobots (red) with lysosomal trackers (green) and DAPI (nucleus, blue) after co-cultured with MDA-MB-231 cells under different conditions and the corresponding fluorescence intensity (FL) profiles. (b) Co-localization of nanorobots (red) with Golgi tracker (green) and DAPI (nucleus, blue) after co-cultured with MDA-MB-231 cells under different conditions and the corresponding fluorescence profiles. From the corresponding fluorescence spectra, it can be seen more clearly that some red fluorescence signals (UHHTN/DOX) partially overlap with green fluorescence (lysosomes, Golgi apparatus) and blue fluorescence (nucleus) and co-localization exists. Scale bar, 50 μm. Lyso, lysosomes; 4',6-diamidino-2-phenylindole, DAPI.

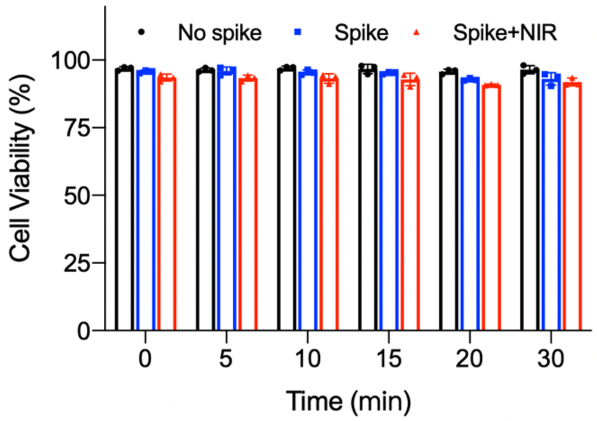

Supplementary Fig. 36. Cell viability during nanoparticles internalization. CCK-8 assay for cells treated with DOX/nanoparticles without nanospikes, spiky nanoparticles, and UHHTN/DOX nanorobots under 1 W·cm^{-2} 980 nm laser irradiation for 0-30 min. Data are presented as the mean ± s.d. (n = 3 replicates). Source data are provided as a Source Data file.

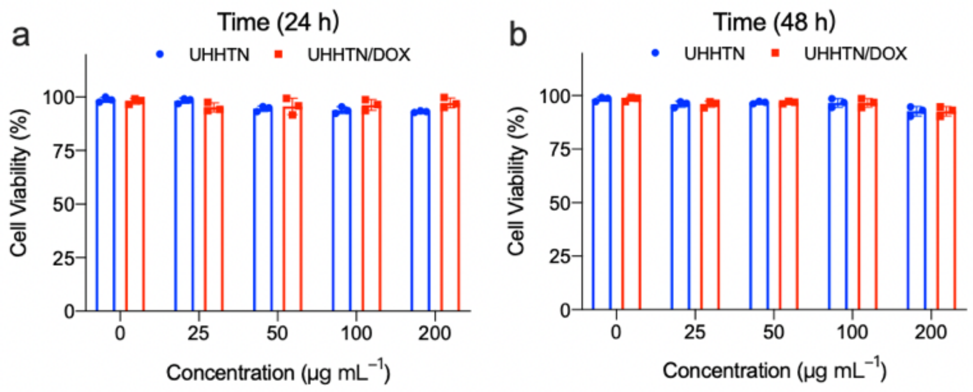

Supplementary Fig. 37. UHHTN/DOX compatibility in MDA-MB-231 tumor cells. CCK-8 assay for cells treated with UHHTN and UHHTN/DOX nanoparticles at

different concentrations for 24 h (a) and 48 h (b). Data are presented as the mean ± s.d. (n = replicates). Source data are provided as a Source Data file.

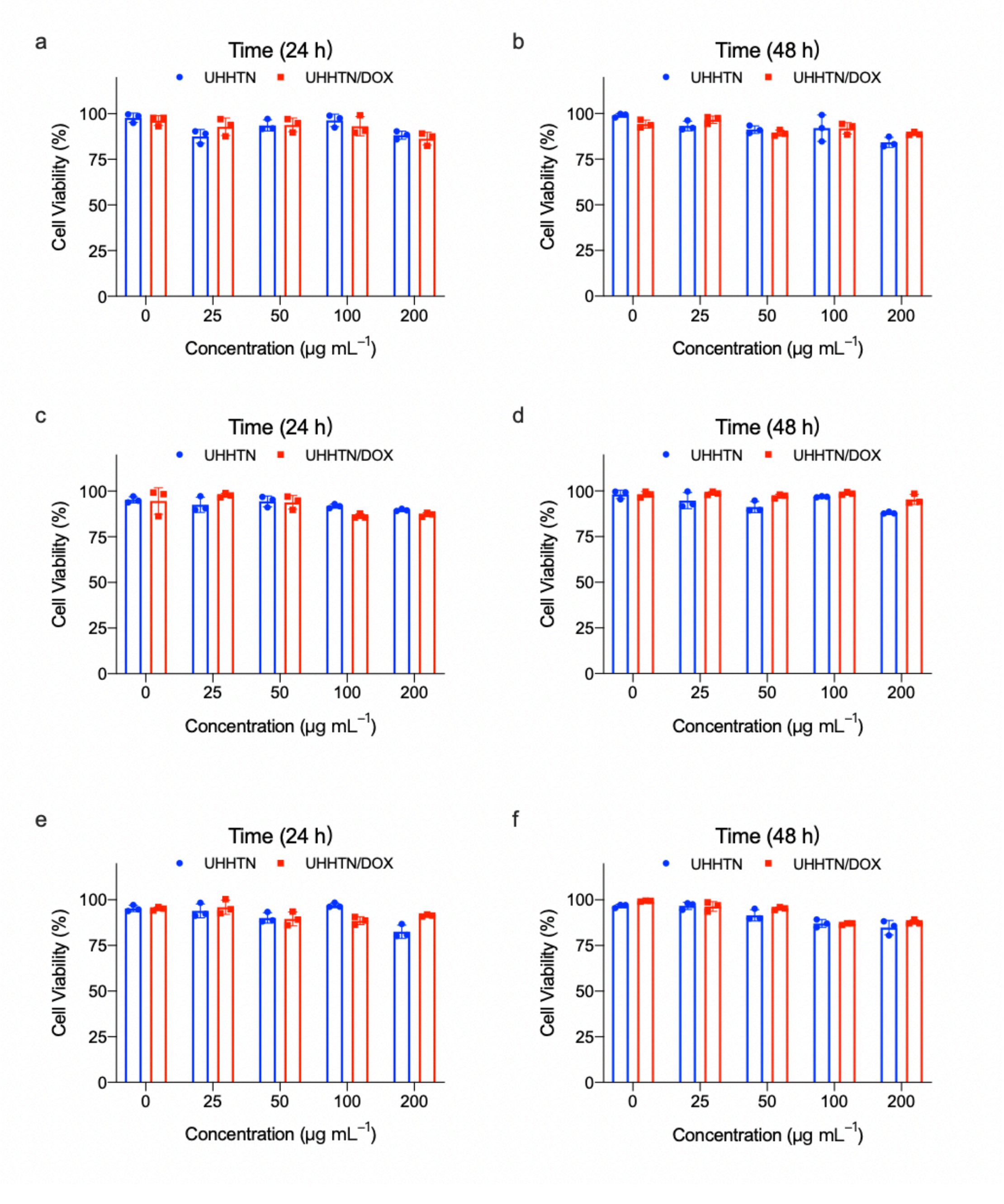

Supplementary Fig. 38. Cytotoxicity of UHHTN and UHHTN/DOX in different tumor cells. Cytotoxicity of UHHTN and UHHTN/DOX in A549 (a-b), PANC-1 (c-d),

B16F10 (e-f) tumor cells at 24 h and 48 h, respectively. Data are presented as the mean ± s.d. (n = 3 replicates). Source data are provided as a Source Data file.

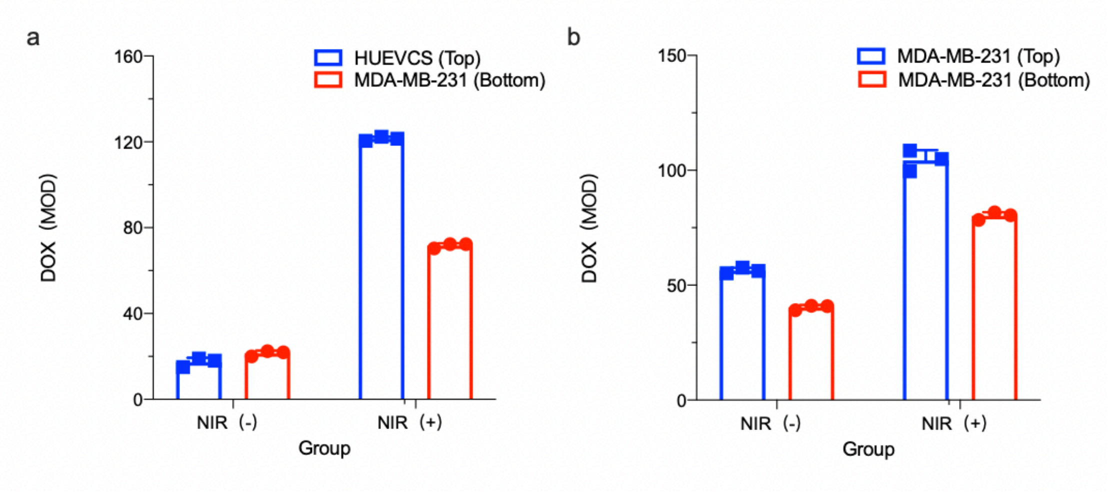

Supplementary Fig. 39. Fluorescence intensity of nanoparticles in different chambers. Fluorescence intensity of transvascular extravasation (a) and fluorescence intensity of intra-tumoral penetration (b). Data are presented as the mean ± s.d. (n = replicates). Source data are provided as a Source Data file.

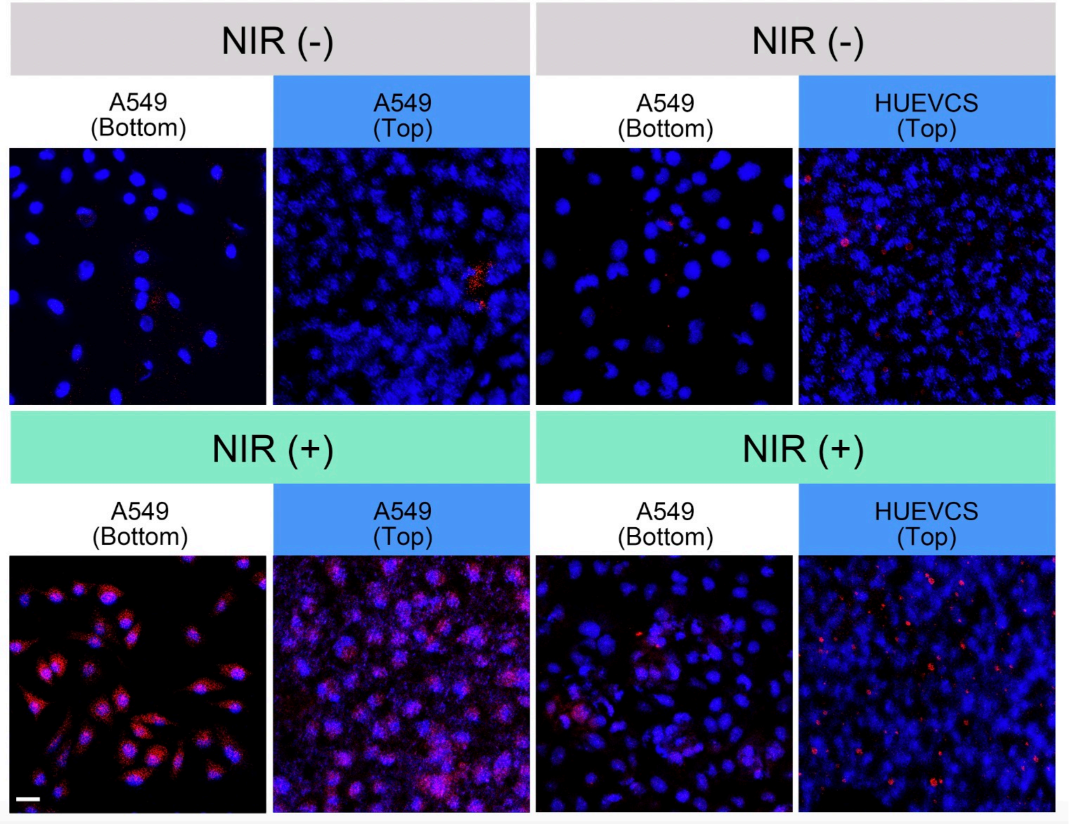

Supplementary Fig. 40. Fluorescence plots Fluorescence plots showing the distribution of UHHTN/DOX nanoparticles in A549 tumor cells in the Transwell chamber with and without laser irradiation. The left side shows the transvascular extravasation results and the right side shows the intra-tumor penetration results. Scale bar, 5 μm.

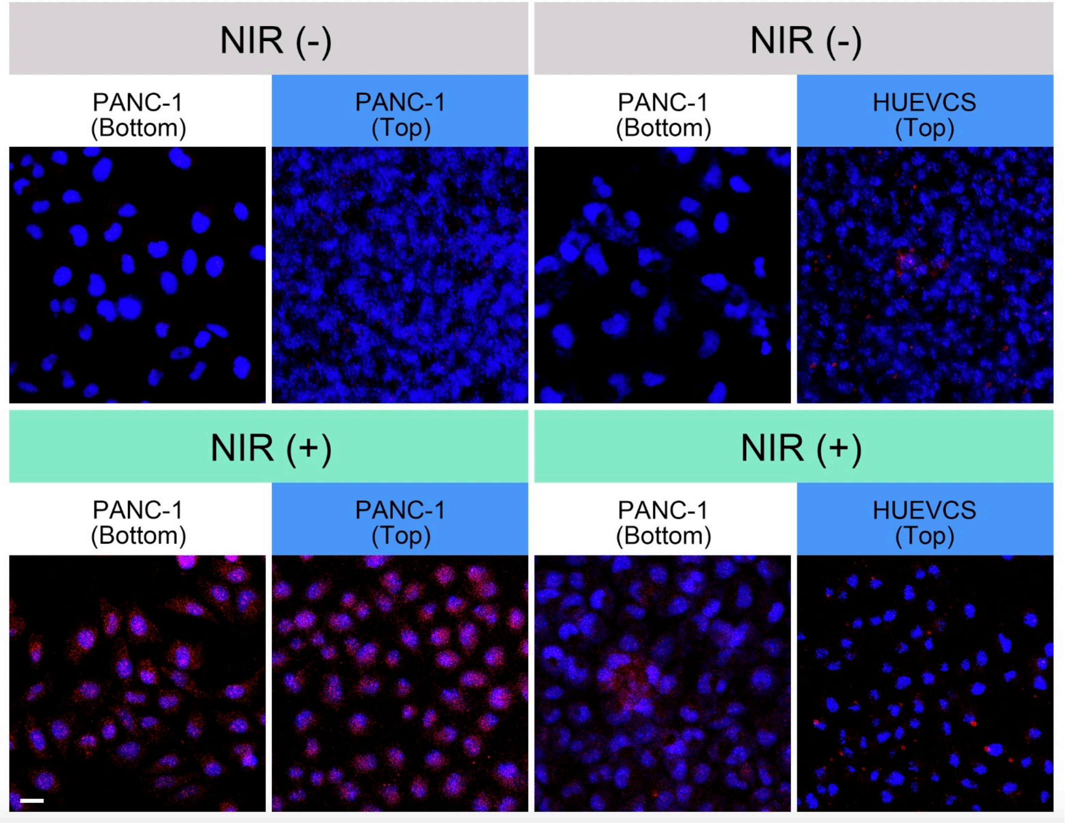

Supplementary Fig. 41. Fluorescence plots showing the distribution of UHHTN/DOX nanoparticles in PANC-1 tumor cells in the Transwell chamber with and without laser irradiation. Scale bar, 25 μm.

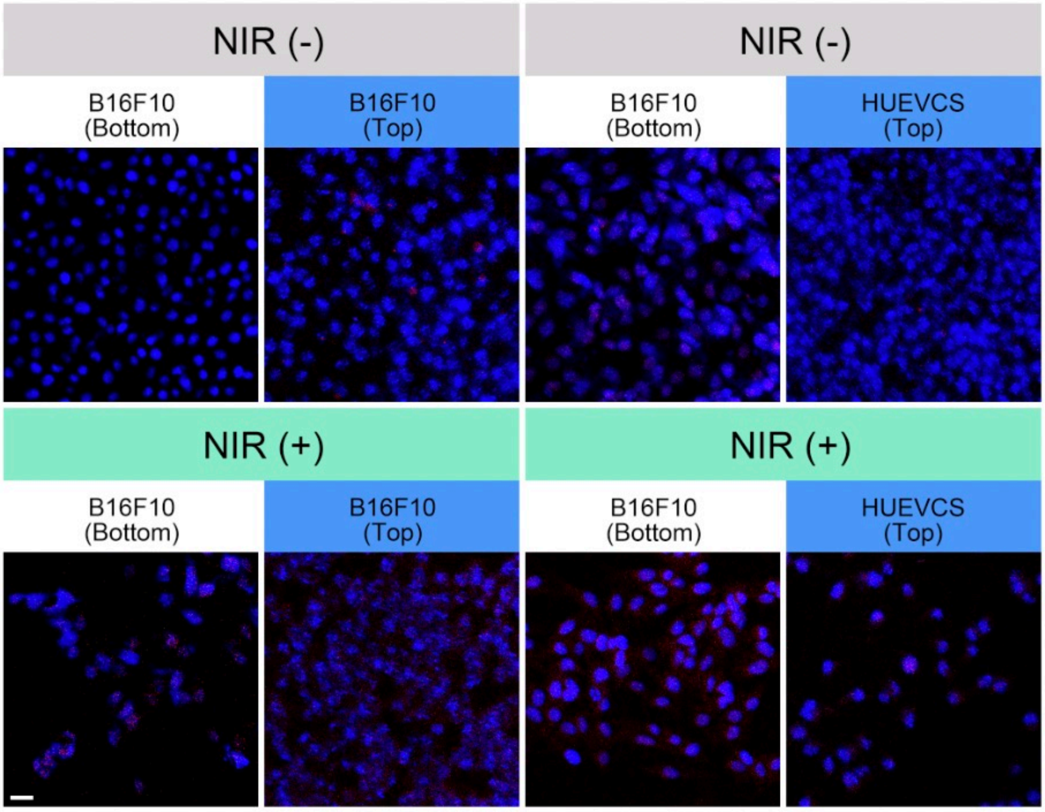

Supplementary Fig. 42. Fluorescence plots showing the distribution of UHHTN/DOX nanoparticles in B16F10 tumor cells in the Transwell chamber with and without laser irradiation. Scale bar, 25 μm.

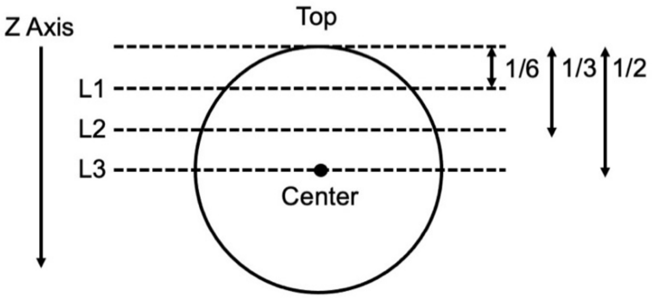

Supplementary Fig. 43. Schematic diagram of the different levels of the 3D tumor spheroid. The Z-axis is used as the long axis of the spheroid, layer 1 (L1) is 1/6 long axis, layer 2 (L2) is 1/3 long axis, and layer 3 (L3) is 1/2.

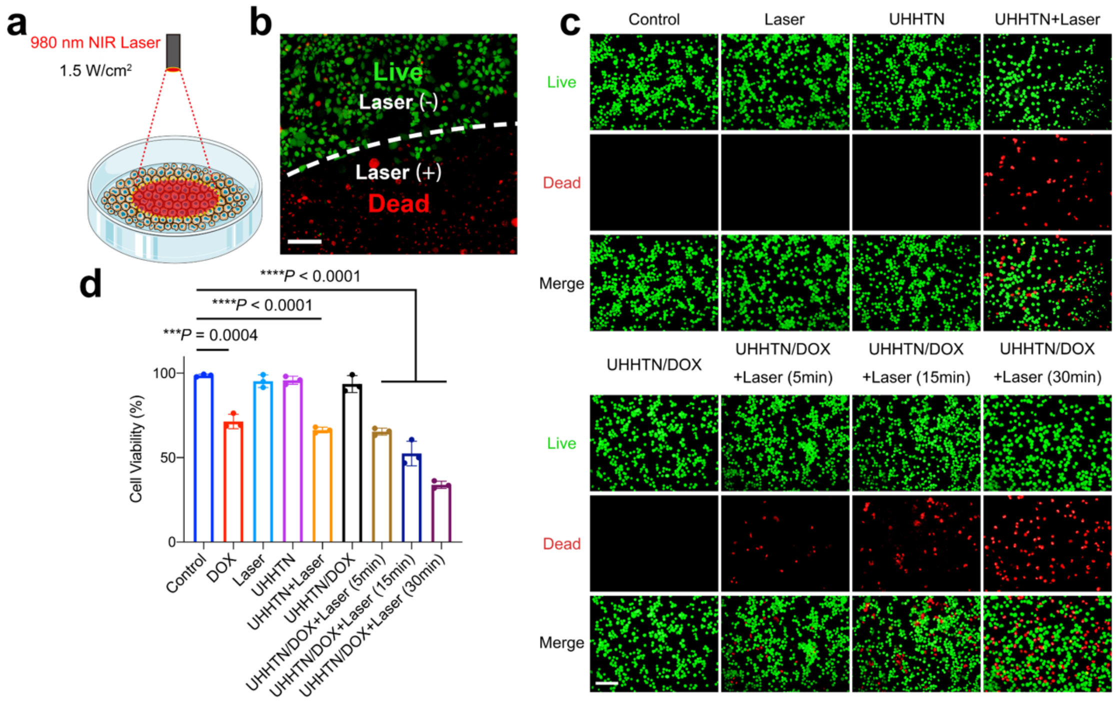

Supplementary Fig. 44. Cytotoxicity of UHHTN/DOX nanorobots in vitro. (a) Schematic diagram of MDA-MB-231 cells irradiated with a 980 nm NIR laser. (b) The boundary areas of the MDA-MB-231 cells with and without laser irradiation are distinguished by dashed lines. Green signal indicates live cells. Red signal indicates dead cells. (c) Live/dead cell staining of MDA-MB-231 cells after different treatments. (d) Cell viability of different treatment groups according to the live/dead assay. Data are presented as the mean ± s.d. Statistical analysis was performed by two-tailed Student's t-test. (n = 3 replicates; ***P < 0.001, ****P < 0.0001). Scale bar, 200 μm. Source data are provided as a Source Data file.

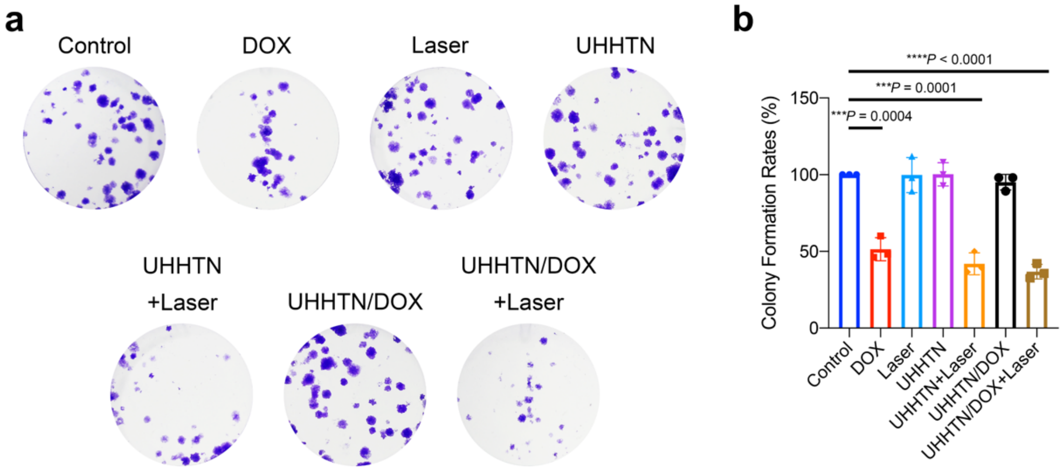

Supplementary Fig. 45. Effect of UHHTN/DOX nanorobots on tumor cell proliferation. (a) Impact of different treatment groups on the proliferation of MDA-MB-231 cells analyzed by colony formation assay. (b) Clone formation rate of different treatment groups. Data are presented as the mean ± s.d. Statistical analysis was performed by two-tailed Student's t-test. (n = 3 replicates; ***P < 0.001, ****P < 0.0001). Source data are provided as a Source Data file.

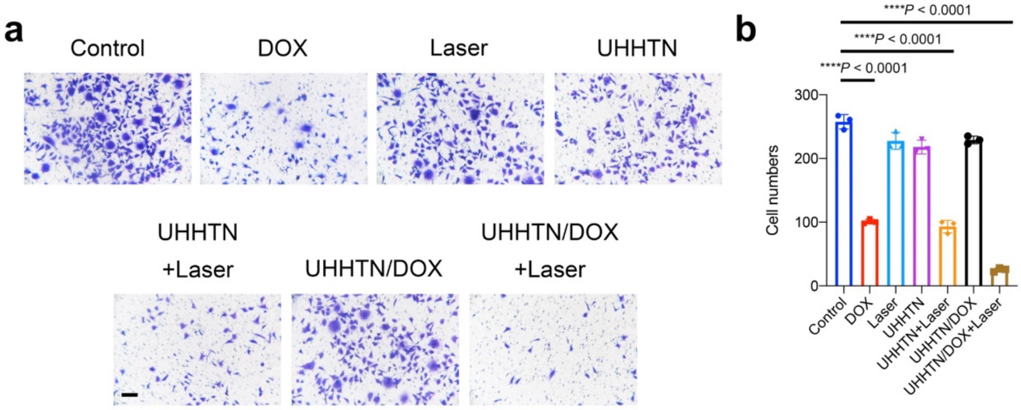

Supplementary Fig. 46. Effect of UHHTN/DOX nanorobots on Transwell migration of tumor cells. (a) Impact of different treatment groups on the migration of MDA-MB-231 cells assessed by Transwell assay without Matrigel. (b) Numbers of migrated cells in different treatment groups. Scale bar = 100 μm. Data are presented as the mean ± s.d. Statistical analysis was performed by two-tailed Student's t-test. (n = 3 replicates; ***P < 0.001, ****P < 0.0001). Source data are provided as a Source Data file.

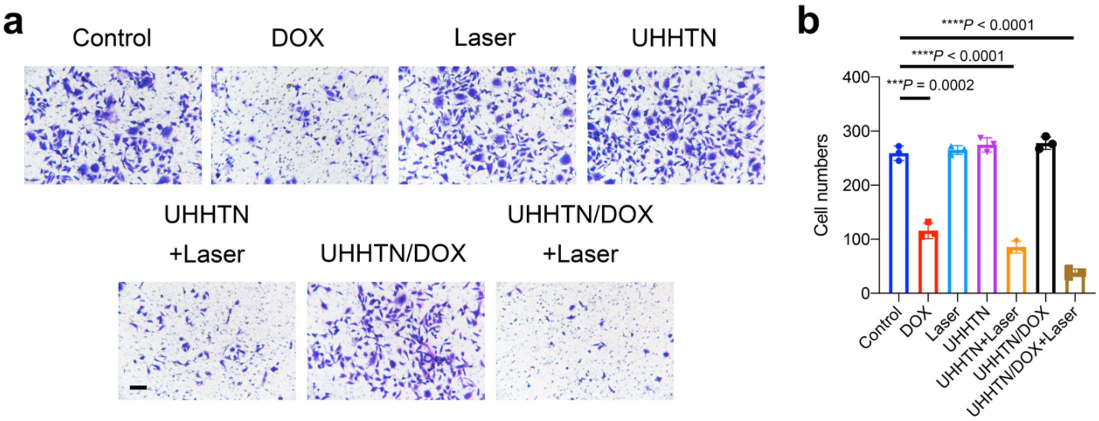

Supplementary Fig. 47. Effect of UHHTN/DOX nanorobots on Transwell invasion of tumor cells. (a) Impact of different treatment groups on MDA-MB-231 cell invasion evaluated by Transwell assay containing Matrigel. (b) Numbers of invasive cells in different treatment groups. Scale bar = 100 μm. Data are presented as the mean ± s.d. Statistical analysis was performed by two-tailed Student's t-test. ($n = 3$ replicates; ***$P < 0.001$, ****$P < 0.0001$). Source data are provided as a Source Data file.

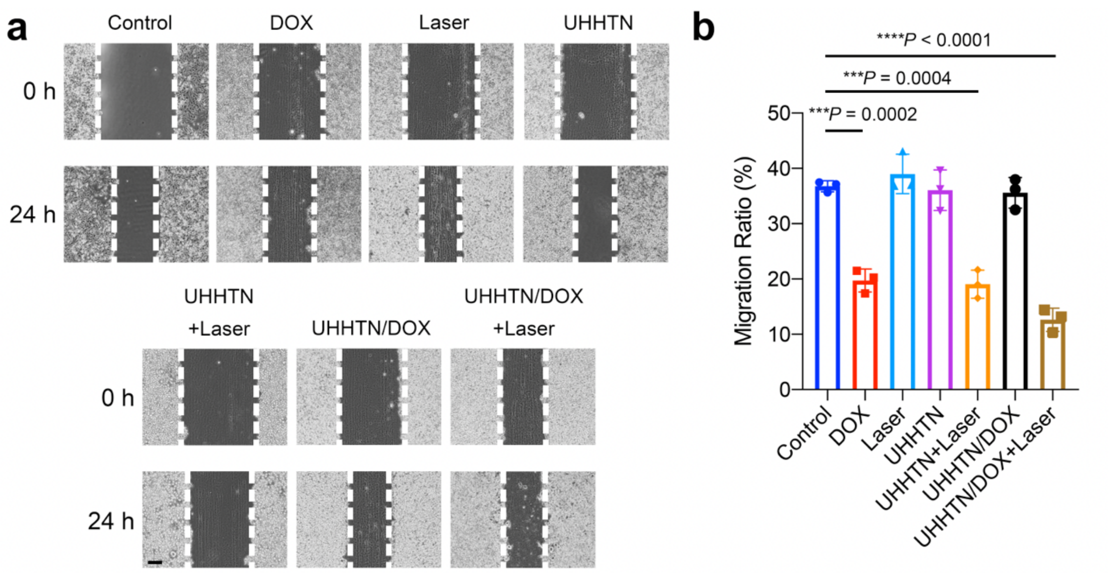

Supplementary Fig. 48. Effect of UHHTN/DOX nanorobots on tumor cell migration. (a) Evaluation of the effects of different treatment groups on the migration of MDA-MB-231 cells at 0 h and 24 h using wound healing assay (Scale bar = 100 μm). (b) Migration ratio of different treatment groups. Data are presented as the mean ± s.d. Statistical analysis was performed by two-tailed Student's t-test. (n = 3 replicates; ***P < 0.001, ****P < 0.0001). Source data are provided as a Source Data file.

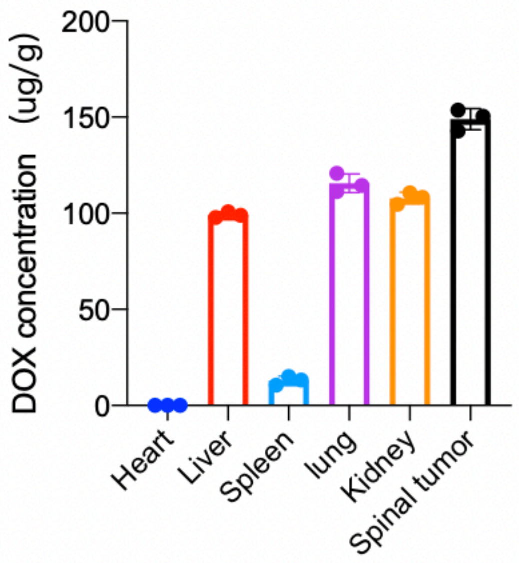

Supplementary Fig. 49. Biodistribution of UHHTN/DOX. DOX concentration in resected major organs and tumors at 24 hours after tail vein injection of UHHTN/DOX at a DOX dosage of 5 mg kg^{-1} in mice. Data are presented as the mean ± s.d. (n = 3 replicates). Source data are provided as a Source Data file.

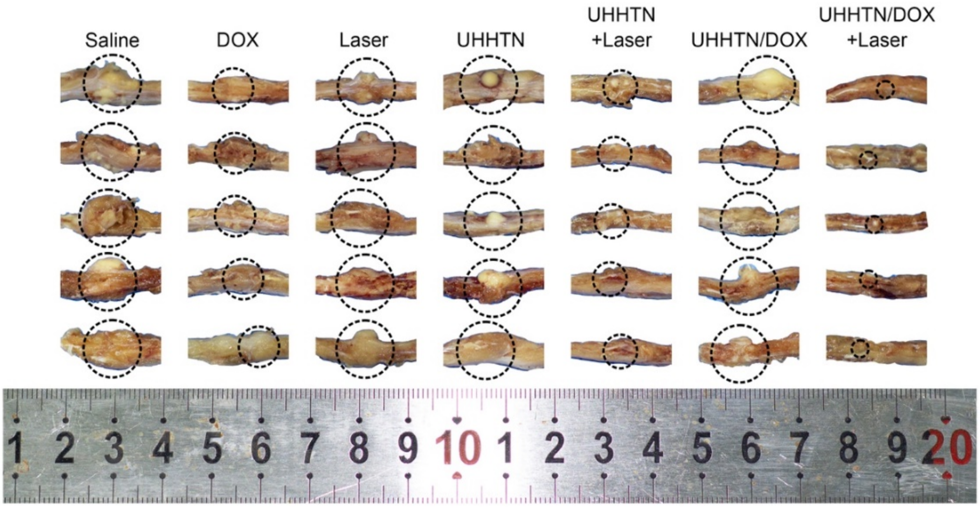

Supplementary Fig. 50. Gross observation of TNBC spinal metastasis specimens 20 days after treatment in different groups at a DOX dosage of 5 mg kg^{-1} once every four days. The spinal tumor was coiled out by a black dummy coil. As seen in the figure, the UHHTN/DOX+Laser treatment group has a higher anti-tumor effect than the UHHTN/DOX treatment and UHHTN+Laser treatment groups.

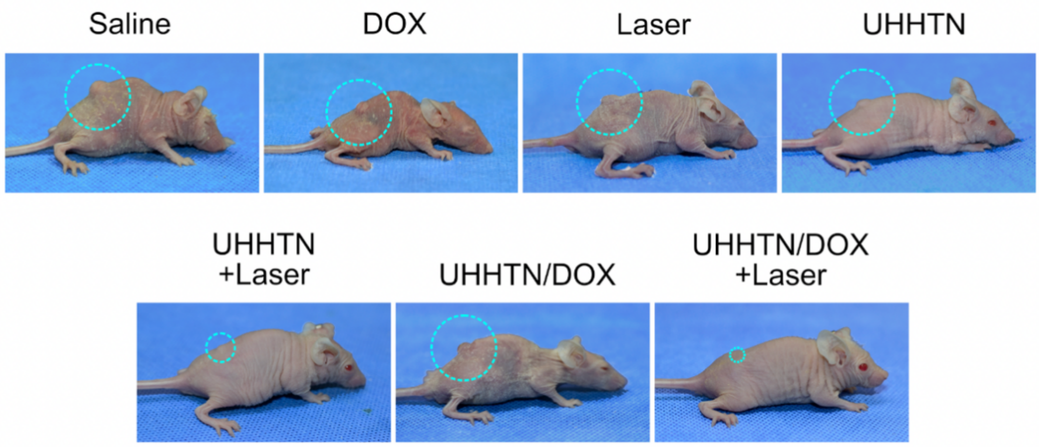

Supplementary Fig. 51. Gross observation imaging of spines in nude mice 20 days after treatment in different groups. Typical images of nude mice with spinal metastases from TNBC at the end of treatment at a DOX dosage of 5 mg kg^{-1} once every four days after 20 days therapy.

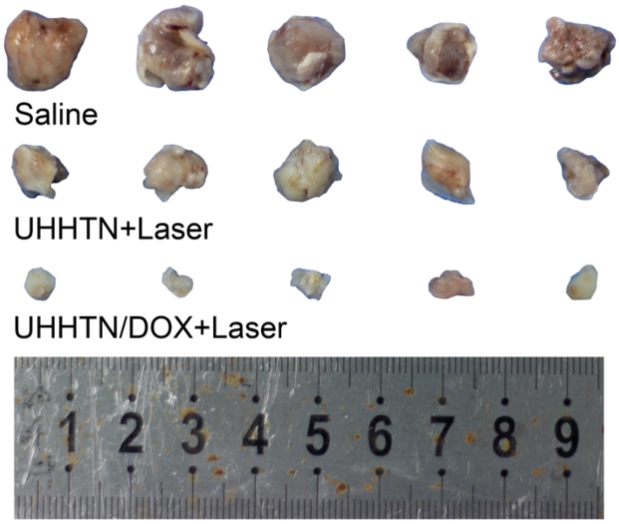

Supplementary Fig. 52. Gross observation of A549-derived subcutaneous tumors. Specimen plots of A549-derived subcutaneous tumors after treatment at a DOX dosage of 5 mg kg^{-1} once every four days after 20 days therapy.

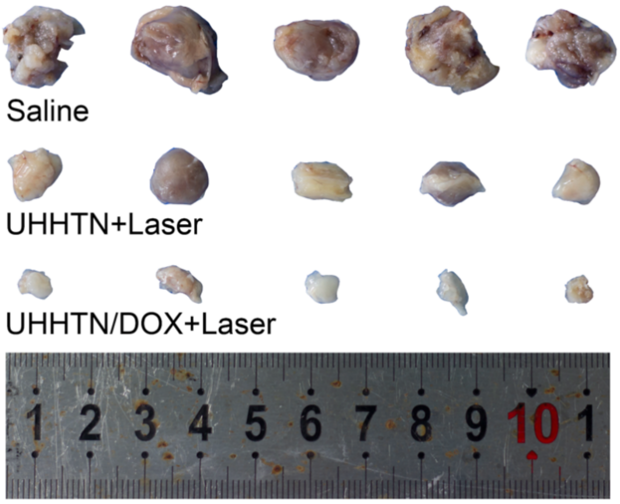

Supplementary Fig. 53. Gross observation of PANC-1-derived subcutaneous tumors. Specimen plots of PANC-1-derived subcutaneous tumors after treatment at a DOX dosage of 5 mg kg^{-1} once every four days after 20 days therapy.

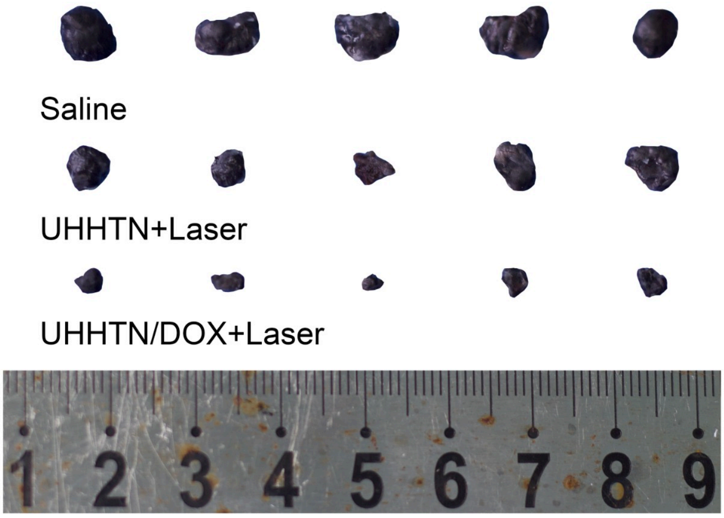

Supplementary Fig. 54. Gross observation of B16F10-derived subcutaneous tumors. Specimen plots of B16F10-derived subcutaneous tumors after treatment at a DOX dosage of 5 mg kg^{-1} once every four days after 20 days therapy.

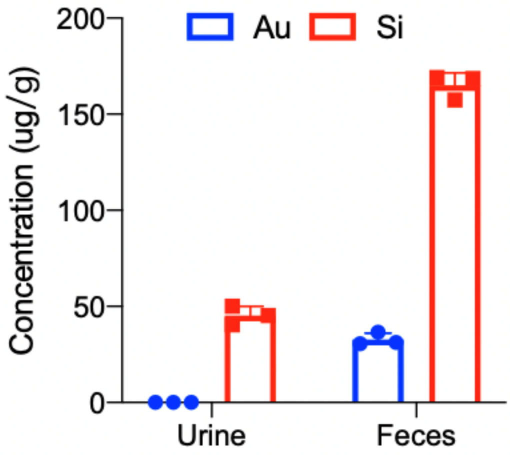

Supplementary Fig. 55. Metabolic pathways of nanoparticles. Quantification of gold and silica in urine and feces by ICP-atomic emission spectrometry after 4 days therapy (UHHTN/DOX nanorobot at a DOX dosage of 5 mg kg^{-1}). Data are presented as the mean ± s.d. (*n* = 3 replicates). Source data are provided as a Source Data file.

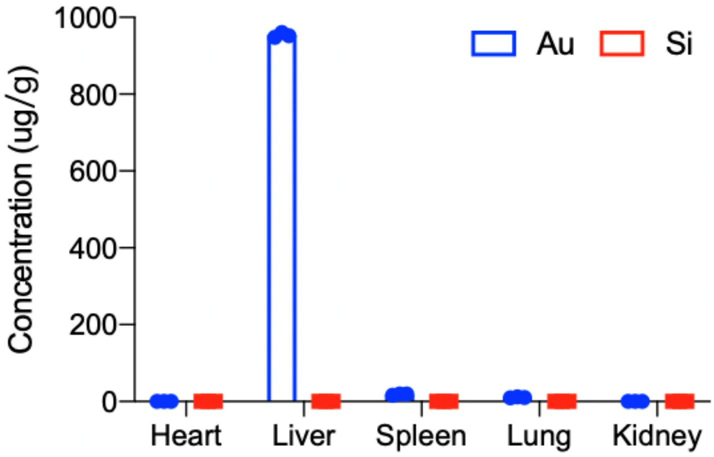

Supplementary Fig. 56. Organ retention after nanoparticles treatments. Quantification of ICP-atomic emission spectra of gold and silica in different organs after 20 days therapy (UHHTN/DOX nanorobot at a DOX dosage of 5 mg kg^{-1} once every four days). Data are presented as the mean ± s.d. (n = 3 replicates). Source data are provided as a Source Data file.

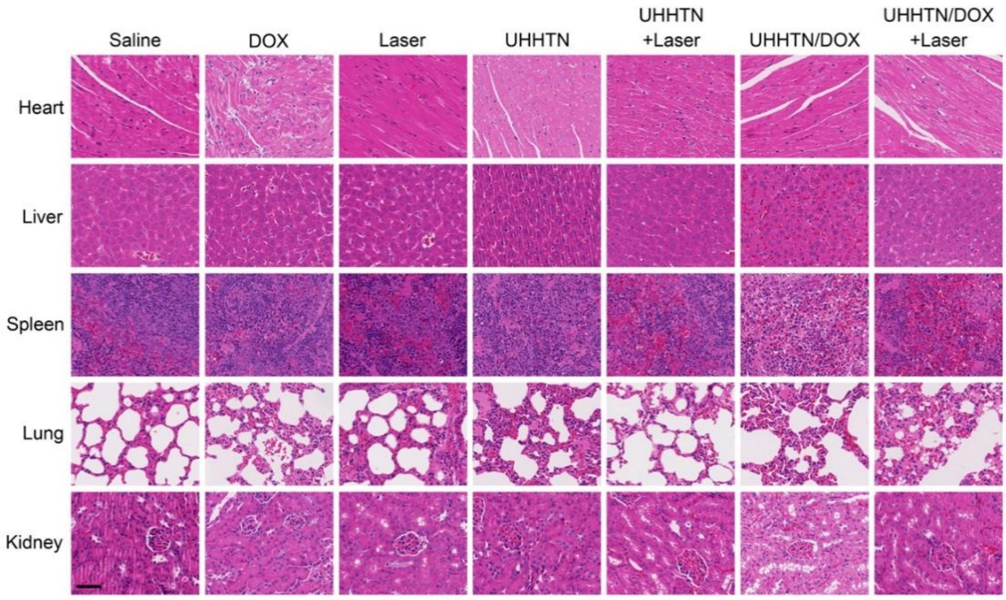

Supplementary Fig. 57. H&E staining of major organs of nude mice with TNBC spinal metastases in different treatment groups at a DOX dosage of 5 mg kg^{-1} once every four days after 20 days therapy. Focal vacuoles caused by cardiotoxic DOX were observed in the hearts of nude mice in the DOX group. In contrast, no significant tissue damage was observed in the major organs in the other treatment groups. Scale bar, 50 μm.

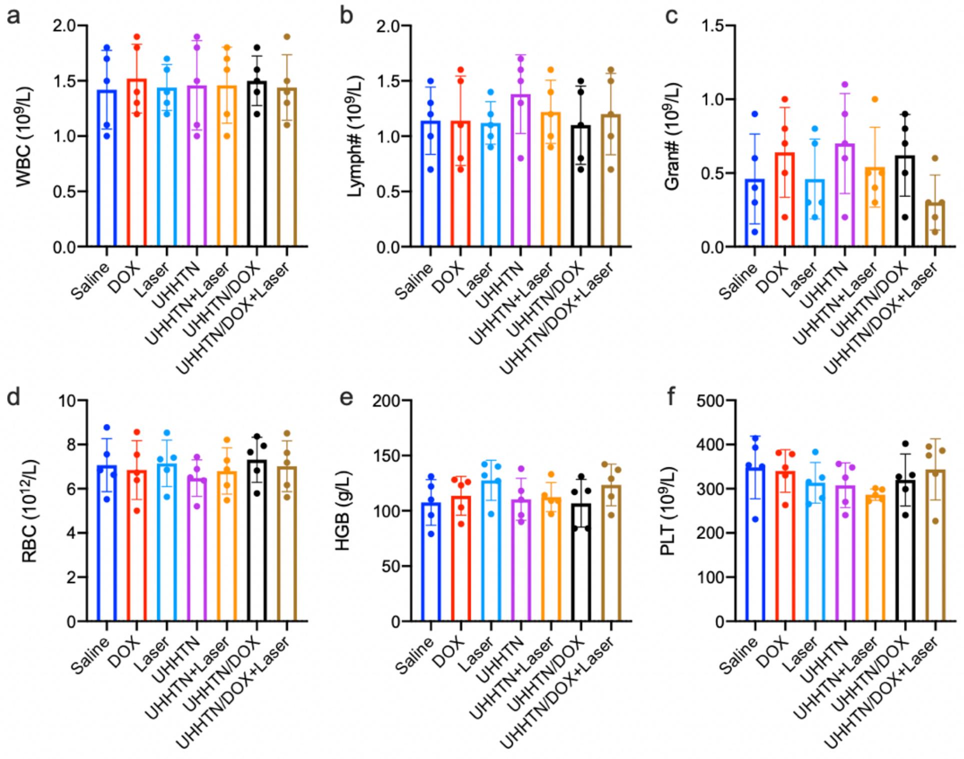

Supplementary Fig. 58. Blood count after nanoparticles treatment. Blood test parameters of white blood cell (WBC), lymphocyte percentage (Lymph#), neutrophil percentage (Gran#), red blood cell (RBC), hemoglobin (HGB) and platelet (PLT) in nude mice with MDA-MB-231-derived spinal metastatic tumors after antitumor treatment at a DOX dosage of 5 mg kg^{-1} once every four days after 20 days therapy. Data are presented as the mean ± s.d. (n = 5 mice per group). Source data are provided as a Source Data file.

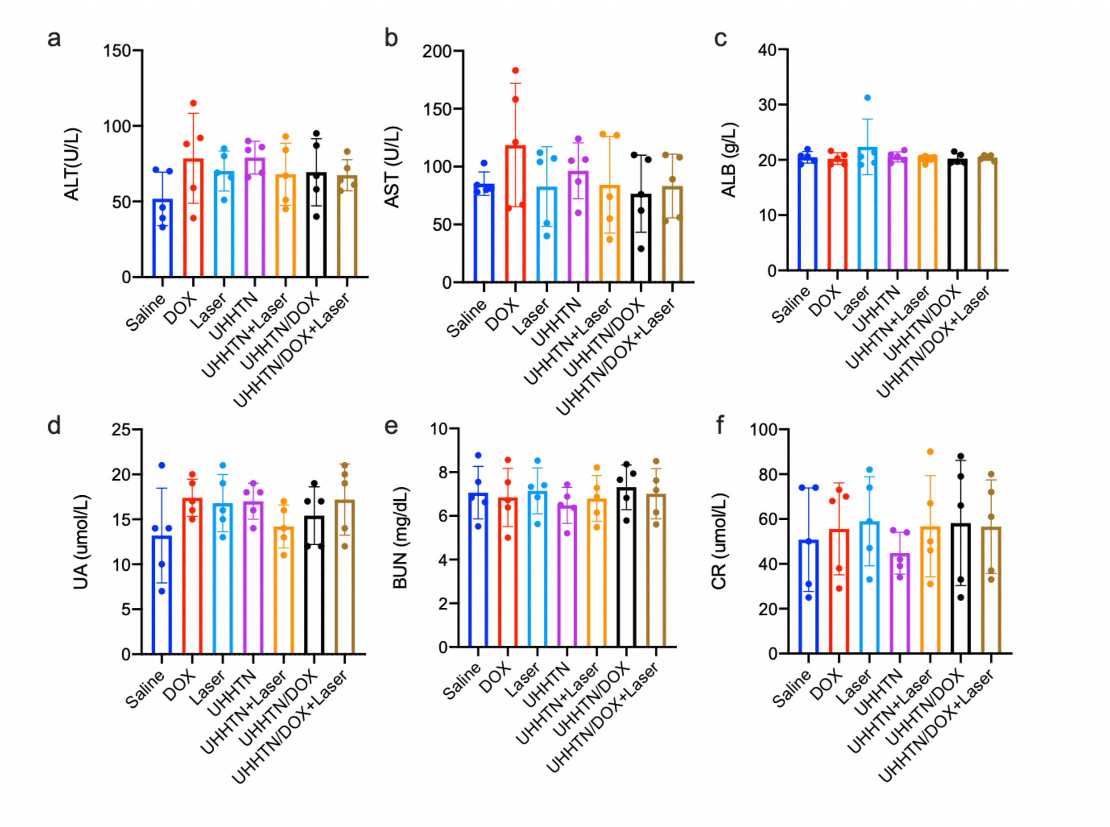

Supplementary Fig. 59. Liver function and kidney function after nanoparticles treatment. Blood test parameters of alanine aminotransferase (ALT), aspartate aminotransferase (AST), albumin (ALB), uric acid (UA), blood urea nitrogen (BUN) and creatinine (CR) in nude mice with MDA-MB-231-derived spinal metastatic tumors after antitumor treatment at a DOX dosage of 5 mg kg^{-1} once every four days after 20 days therapy. Data are presented as the mean ± s.d. (n = 5 mice per group). Source data are provided as a Source Data file.

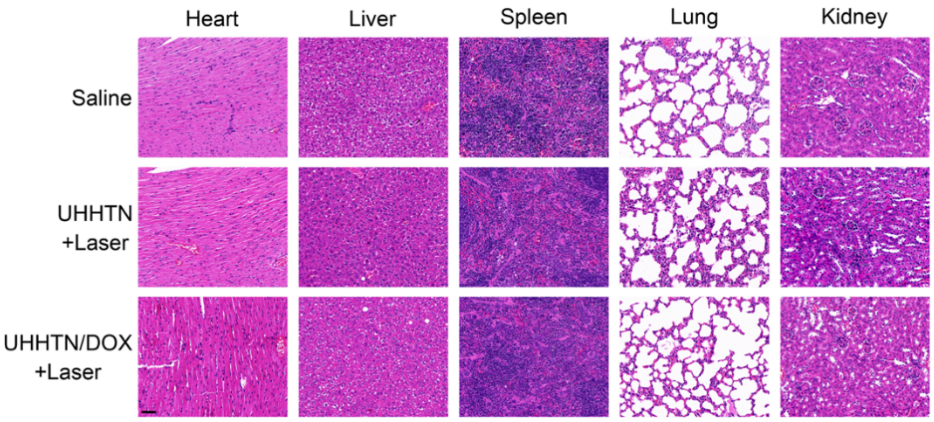

Supplementary Fig. 60. Organ toxicity after nanoparticles therapy in A549-derived subcutaneous tumor models. H&E staining of different organs in A549-derived subcutaneous tumor models with different treatment at a DOX dosage of 5 mg kg^{-1} once every four days after 20 days therapy. Scale bar, 20 μm.

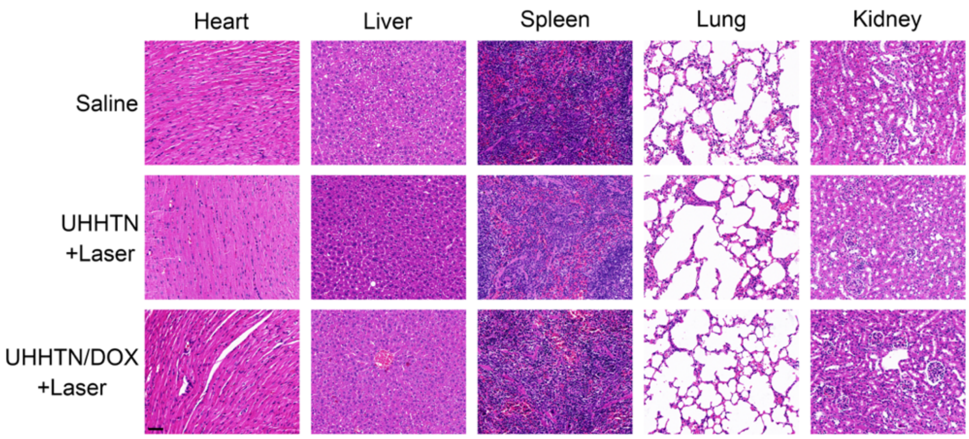

Supplementary Fig. 61. Organ toxicity after nanoparticles therapy in PANC-1-derived subcutaneous tumor models. H&E staining of different organs in PANC-1-derived subcutaneous tumor models with different treatment at a DOX dosage of 5 mg kg^{-1} once every four days after 20 days therapy. Scale bar, 20 μm.

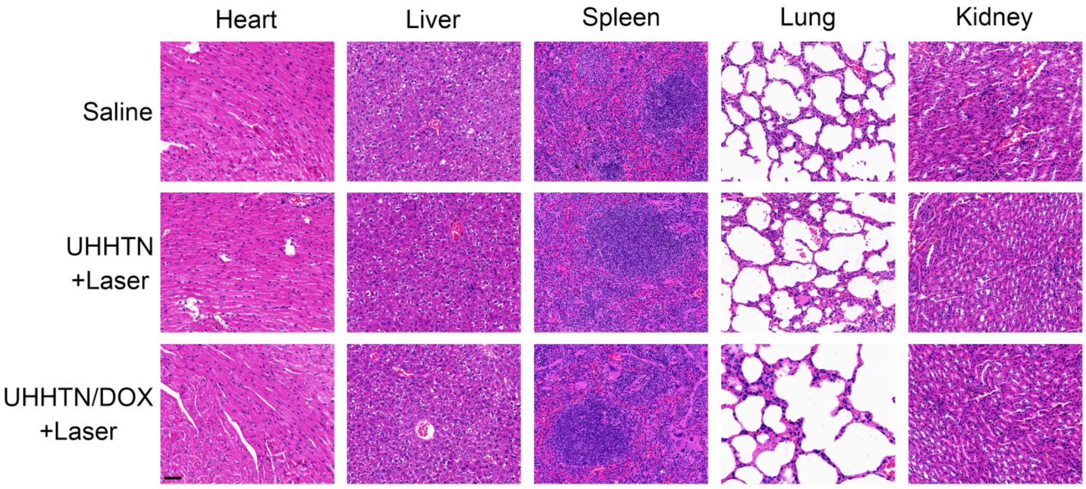

Supplementary Fig. 62. Organ toxicity after nanoparticles therapy in B16F10-derived subcutaneous tumor models. H&E staining of different organs in B16F10-derived subcutaneous tumor models with different treatment at a DOX dosage of 5 mg kg^{-1} once every four days after 20 days therapy. Scale bar, 20 μm.

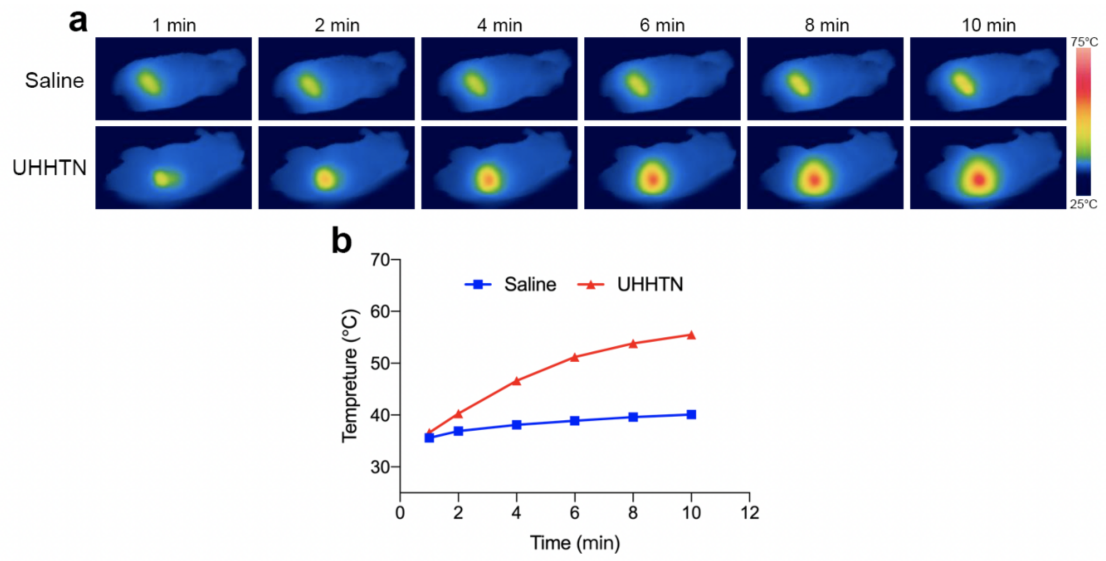

Supplementary Fig. 63. Photothermal effect of nanoparticles in vivo. (a) Thermograms and (b) temperature variation of spinal tumors with laser irradiation time (980 nm, 1.5 W·cm^{-2}) after saline and UHHTNs treatment after 10 minutes.

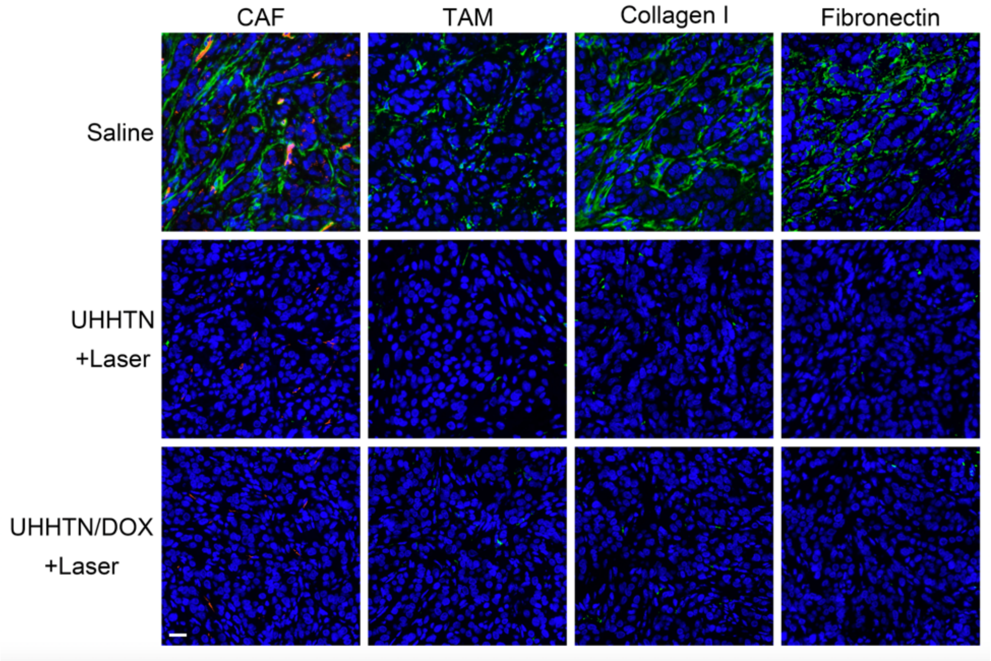

Supplementary Fig. 64. The photothermal effect mediated TSM remodeling of UHHTN/DOX nanoparticles in A549-derived subcutaneous tumors. The photothermal effect mediated TSM remodeling of UHHTN/DOX nanorobot in A549-derived subcutaneous tumors with different treatment at a DOX dosage of 5 mg kg^{-1} once every four days after 20 days therapy (green, α-SMA, F4/80, Collagen I, Fibronectin; red, CD31; blue, DAPI; Scale bar = 20 μm). These fluorescent signals of CAF, TAM, collagen I and fibronectin were significantly attenuated in UHHTN+Laser and UHHTN/DOX+Laser treated subcutaneous tumors compared to saline treated subcutaneous tumors.

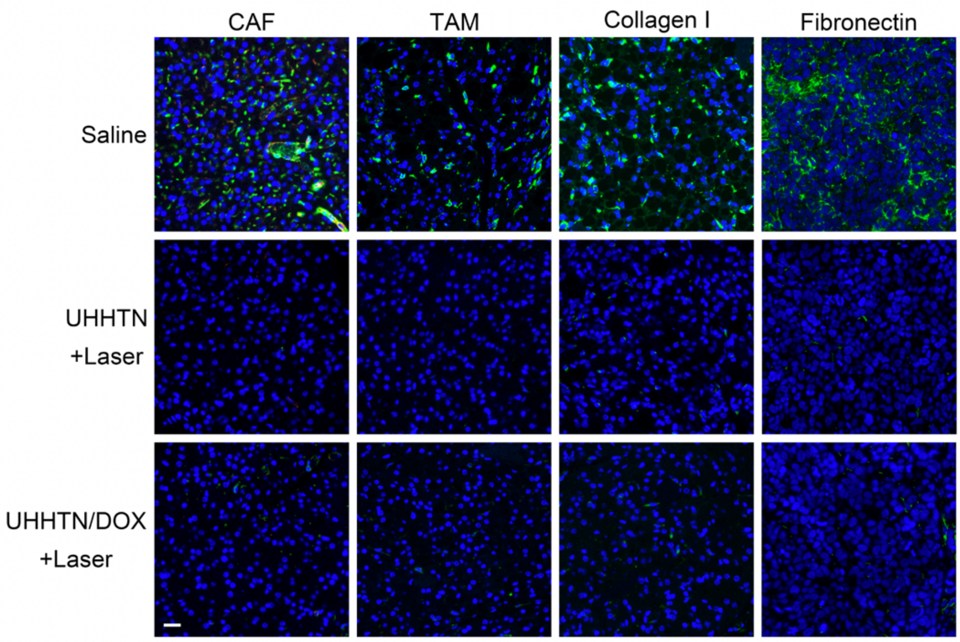

Supplementary Fig. 65. The photothermal effect mediated TSM remodeling of UHHTN/DOX nanoparticles in PANC-1-derived subcutaneous tumors. The photothermal effect mediated TSM remodeling of UHHTN/DOX nanorobot in PANC-1-derived subcutaneous tumors with different treatment at a DOX dosage of 5 mg kg^{-1} once every four days after 20 days therapy (green, α-SMA, F4/80, Collagen I, Fibronectin; red, CD31; blue, DAPI; Scale bar = 20 μm). These fluorescent signals of CAF, TAM, collagen I and fibronectin were significantly attenuated in UHHTN+Laser and UHHTN/DOX+Laser treated subcutaneous tumors compared to saline treated subcutaneous tumors.

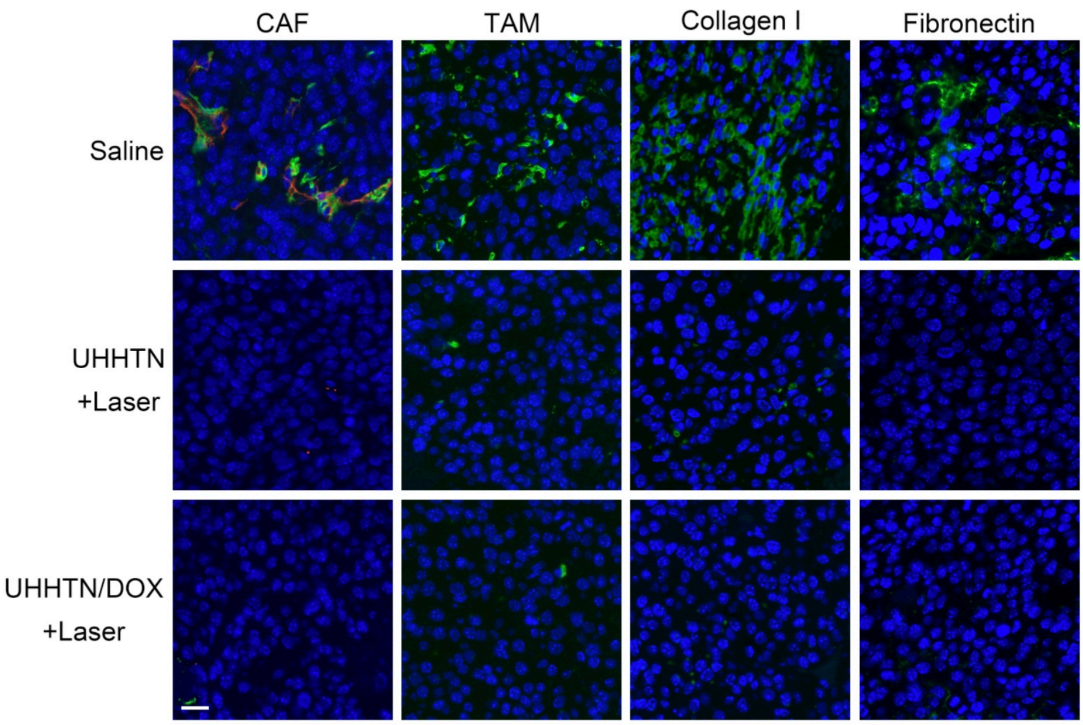

Supplementary Fig. 66. The photothermal effect mediated TSM remodeling of UHHTN/DOX nanorobot in B16F10-derived subcutaneous tumors with different treatment at a DOX dosage of 5 mg kg^{-1} once every four days after 20 days therapy (green, α-SMA, F4/80, Collagen I, Fibronectin; red, CD31; blue, DAPI; Scale bar = 20 μm). These fluorescent signals of CAF, TAM, collagen I and fibronectin were significantly attenuated in UHHTN+Laser and UHHTN/DOX+Laser treated subcutaneous tumors compared to saline treated subcutaneous tumors.

4. References

1. Dunderdale, G., Ebbens, S., Fairclough, P.& Howse, J. Importance of Particle Tracking and Calculating the Mean-Squared Displacement in Distinguishing Nanopropulsion from Other Processes. *Langmuir* **28**, 10997-11006 (2012).
2. Jiang, H.-R., Yoshinaga, N.& Sano, M. Active Motion of a Janus Particle by Self-Thermophoresis in a Defocused Laser Beam. *Phys. Rev. Lett.* **105**, 268302 (2010).
