# Supplementary material for: Site-selective superassembly of biomimetic nanorobots enabling deep penetration into tumor with stiff stroma
Source: Nat Commun. 2023 Aug 2;14:4628. doi: 10.1038/s41467-023-40300-2 (PMC10397308; doi:10.1038/s41467-023-40300-2)
Supplement: Supplementary file 3 — Description of Additional Supplementary Information Files [file 41467_2023_40300_MOESM3_ESM.pdf]

Description of Additional Supplementary Information Files

File Name: Supplementary Video 1.

Description: UHHTN nanoparticle movement at 0 $W \cdot cm^{-2}$ laser power (AVI).

File Name: Supplementary Video 2.

Description: UHHTN nanorobot movement at 0.5 $W \cdot cm^{-2}$ laser power (AVI).

File Name: Supplementary Video 3.

Description: UHHTN nanorobot movement at 1.0 $W \cdot cm^{-2}$ laser power (AVI).

File Name: Supplementary Video 4.

Description: UHHTN nanorobot movement at 1.5 $W \cdot cm^{-2}$ laser power (AVI).

File Name: Supplementary Video 5.

UHHTN nanorobot movement at 2.0 $W \cdot cm^{-2}$ laser power (AVI)
