# Supplementary material for: Site-selective superassembly of biomimetic nanorobots enabling deep penetration into tumor with stiff stroma
Source: Nat Commun. 2023 Aug 2;14:4628. doi: 10.1038/s41467-023-40300-2 (PMC10397308; doi:10.1038/s41467-023-40300-2)
Supplement: Supplementary file 9 — Reporting Summary [file 41467_2023_40300_MOESM9_ESM.pdf]

nature portfolio

Corresponding author(s): Biao Kong, Libo Jiang

Last updated by author(s): YYYY-MM-DD

Reporting Summary

Nature Portfolio wishes to improve the reproducibility of the work that we publish. This form provides structure for consistency and transparency in reporting. For further information on Nature Portfolio policies, see our Editorial Policies and the Editorial Policy Checklist.

Statistics

For all statistical analyses, confirm that the following items are present in the figure legend, table legend, main text, or Methods section.

n/a	Confirmed	
☐	☒	The exact sample size (*n*) for each experimental group/condition, given as a discrete number and unit of measurement
☐	☒	A statement on whether measurements were taken from distinct samples or whether the same sample was measured repeatedly
☐	☒	The statistical test(s) used AND whether they are one- or two-sided<br>*Only common tests should be described solely by name; describe more complex techniques in the Methods section.*
☒	☐	A description of all covariates tested
☒	☐	A description of any assumptions or corrections, such as tests of normality and adjustment for multiple comparisons
☐	☒	A full description of the statistical parameters including central tendency (e.g. means) or other basic estimates (e.g. regression coefficient) AND variation (e.g. standard deviation) or associated estimates of uncertainty (e.g. confidence intervals)
☐	☒	For null hypothesis testing, the test statistic (e.g. *F*, *t*, *r*) with confidence intervals, effect sizes, degrees of freedom and *P* value noted<br>*Give P values as exact values whenever suitable.*
☒	☐	For Bayesian analysis, information on the choice of priors and Markov chain Monte Carlo settings
☒	☐	For hierarchical and complex designs, identification of the appropriate level for tests and full reporting of outcomes
☒	☐	Estimates of effect sizes (e.g. Cohen's *d*, Pearson's *r*), indicating how they were calculated

Our web collection on statistics for biologists contains articles on many of the points above.

Software and code

Policy information about availability of computer code

Data collection	Olympus FV31S-SW software 2.3.2, Living Image 4.4, ParaVision software 4.1, NRecon software 2.1, SPSS software 25.0, Microsoft Excel 2021.
Data analysis	Softwares used for analysis include GraphPad Prism 8.0, Image J 2.3.0, Living Image 4.4, RadiAnt DICOM Viewer software 2020.2.2, CTAn software 1.15, SPSS software 25.0, Microsoft Excel 2021.

For manuscripts utilizing custom algorithms or software that are central to the research but not yet described in published literature, software must be made available to editors and reviewers. We strongly encourage code deposition in a community repository (e.g. GitHub). See the Nature Portfolio guidelines for submitting code & software for further information.

Data

Policy information about availability of data

All manuscripts must include a data availability statement. This statement should provide the following information, where applicable:

- Accession codes, unique identifiers, or web links for publicly available datasets
- A description of any restrictions on data availability
- For clinical datasets or third party data, please ensure that the statement adheres to our policy

The experimental data supporting the findings of this study are available within the article and the Supplementary Information. Extra data are available from the corresponding authors upon reasonable request. Source data are provided with this paper.

Human research participants

Policy information about studies involving human research participants and Sex and Gender in Research.

Reporting on sex and gender	*Use the terms sex (biological attribute) and gender (shaped by social and cultural circumstances) carefully in order to avoid confusing both terms. Indicate if findings apply to only one sex or gender; describe whether sex and gender were considered in study design whether sex and/or gender was determined based on self-reporting or assigned and methods used. Provide in the source data disaggregated sex and gender data where this information has been collected, and consent has been obtained for sharing of individual-level data; provide overall numbers in this Reporting Summary. Please state if this information has not been collected. Report sex- and gender-based analyses where performed, justify reasons for lack of sex- and gender-based analysis.*
Population characteristics	*Describe the covariate-relevant population characteristics of the human research participants (e.g. age, genotypic information, past and current diagnosis and treatment categories). If you filled out the behavioural & social sciences study design questions and have nothing to add here, write "See above."*
Recruitment	*Describe how participants were recruited. Outline any potential self-selection bias or other biases that may be present and how these are likely to impact results.*
Ethics oversight	*Identify the organization(s) that approved the study protocol.*

Note that full information on the approval of the study protocol must also be provided in the manuscript.

Field-specific reporting

Please select the one below that is the best fit for your research. If you are not sure, read the appropriate sections before making your selection.

☒ Life sciences ☐ Behavioural & social sciences ☐ Ecological, evolutionary & environmental sciences

For a reference copy of the document with all sections, see nature.com/documents/nr-reporting-summary-flat.pdf

Life sciences study design

All studies must disclose on these points even when the disclosure is negative.

Sample size	For in vitro or ex vivo studies, the sample size we used n=3 as the minimum value to obtain statistically significant and significant results. Similarly, for in vivo studies, n=5 was sufficient to detect significant biological differences with good reproducibility. Detailed information on sample sizes for all experiments is provided in the Methods section and in the figure legends.
Data exclusions	No data were excluded.
Replication	The experimental data in this paper were all performed at least 3 times and more, as shown in the figure caption. The replication experiments were successful and achieved similar results.
Randomization	In vitro cells and mice used for in vivo experiments were randomly assigned to treatment groups.
Blinding	During the data collection and analysis process, the researchers were unaware of the assigned groupings.

Reporting for specific materials, systems and methods

We require information from authors about some types of materials, experimental systems and methods used in many studies. Here, indicate whether each material, system or method listed is relevant to your study. If you are not sure if a list item applies to your research, read the appropriate section before selecting a response.

Materials & experimental systems

n/a	Involved in the study	
☐	☒	Antibodies
☐	☒	Eukaryotic cell lines
☒	☐	Palaeontology and archaeology
☐	☒	Animals and other organisms
☒	☐	Clinical data
☒	☐	Dual use research of concern

Methods

n/a	Involved in the study	
☒	☐	ChIP-seq
☒	☐	Flow cytometry
☒	☐	MRI-based neuroimaging

Antibodies

Antibodies used	From Cell Signaling Technology:<br>F4/80 (D2S9R) XP® Rabbit mAb, #70076s, 20200915, dilution of 1/500<br>COL1A1 Antibody, #84336s, 20190817, dilution of 1/1000<br>From Abcam:<br>Anti-Fibronectin antibody, #ab2413, 20210305, dilution of1/1000<br>Anti-alpha smooth muscle Actin antibody, #ab7817, 20190526, dilution of 1/500<br>Anti-CD31 antibody, #ab182981, 20200618, dilution of 1/2000
Validation	All antibodies were verified by the supplier and each lot has been quality tested. Validation statements are shown on the manufacturer's website.

Eukaryotic cell lines

Policy information about cell lines and Sex and Gender in Research

Cell line source(s)	MDA-MB-231, A549, PANC-1, B16F10 and human umbilical vein cells (HUVECs) were acquired from the Institute of Biochemistry and Cell Biology, Chinese Academy of Sciences, Shanghai, China.
Authentication	Cell lines were authenticated by Short Tandem Repeat (STR) test.
Mycoplasma contamination	No mycoplasma contamination was found in the assay experiments for all cells.
Commonly misidentified lines<br>(See ICLAC register)	None of the cell lines used in this study were present in ICLAC.

Animals and other research organisms

Policy information about studies involving animals; ARRIVE guidelines recommended for reporting animal research, and Sex and Gender in Research

Laboratory animals	BALB/c nude mice and C57 mice (4-6 weeks, female, with an average of 18 g) were purchased from Shanghai JieSiJie Laboratory Animals Co. LTD. Mice were housed at an ambient temperature of 22°C and 45% humidity with a diurnal cycle of 14/10 (on at 6:00 and off at 20:00).
Wild animals	This study did not involve wild animals.
Reporting on sex	The model system was a spinal metastasis model of breast cancer, and males do not have the possibility of developing breast cancer, so only female rats were selected. To ensure the consistency of the animal model, female rats were subsequently used in lung cancer, pancreatic cancer, and melanoma.
Field-collected samples	This study did not involve biological samples collected from the field.
Ethics oversight	All animal experiment protocols were performed in accordance with the Guidelines for the Care and Use of Laboratory Animals approved by the Animal Experimentation Ethics Committee of Zhongshan Hospital, Fudan University.

Note that full information on the approval of the study protocol must also be provided in the manuscript.
